# Late-pregnancy dysglycemia in obese pregnancies after negative testing for gestational diabetes and risk of future childhood overweight: An interim analysis from a longitudinal mother–child cohort study

**DOI:** 10.1371/journal.pmed.1002681

**Published:** 2018-10-29

**Authors:** Delphina Gomes, Rüdiger von Kries, Maria Delius, Ulrich Mansmann, Martha Nast, Martina Stubert, Lena Langhammer, Nikolaus A. Haas, Heinrich Netz, Viola Obermeier, Stefan Kuhle, Lesca M. Holdt, Daniel Teupser, Uwe Hasbargen, Adelbert A. Roscher, Regina Ensenauer

**Affiliations:** 1 Department of General Pediatrics, Neonatology and Pediatric Cardiology, Division of Experimental Pediatrics and Metabolism, University Children's Hospital, Faculty of Medicine, Heinrich Heine University Düsseldorf, Düsseldorf, Germany; 2 Institute of Social Paediatrics and Adolescent Medicine, Division of Epidemiology, Faculty of Medicine, Ludwig-Maximilians-Universität München, Munich, Germany; 3 Department of Obstetrics and Gynecology, University Hospital, Ludwig-Maximilians-Universität München, Munich, Germany; 4 Institute for Medical Information Processing, Biometry, and Epidemiology (IBE), Faculty of Medicine, Ludwig-Maximilians-Universität München, Munich, Germany; 5 Division of Pediatric Cardiology and Intensive Care, University Hospital, Ludwig-Maximilians-Universität München, Munich, Germany; 6 Institute of Laboratory Medicine, University Hospital, Ludwig-Maximilians-Universität München, Munich, Germany; 7 Department of Pediatrics, University Hospital, Ludwig-Maximilians-Universität München, Munich, Germany; University of Manchester, UNITED KINGDOM

## Abstract

**Background:**

Maternal pre-conception obesity is a strong risk factor for childhood overweight. However, prenatal mechanisms and their effects in susceptible gestational periods that contribute to this risk are not well understood. We aimed to assess the impact of late-pregnancy dysglycemia in obese pregnancies with negative testing for gestational diabetes mellitus (GDM) on long-term mother–child outcomes.

**Methods and findings:**

The prospective cohort study Programming of Enhanced Adiposity Risk in Childhood–Early Screening (PEACHES) (*n =* 1,671) enrolled obese and normal weight mothers from August 2010 to December 2015 with trimester-specific data on glucose metabolism including GDM status at the end of the second trimester and maternal glycated hemoglobin (HbA_1c_) at delivery as a marker for late-pregnancy dysglycemia (HbA_1c_ ≥ 5.7% [39 mmol/mol]). We assessed offspring short- and long-term outcomes up to 4 years, and maternal glucose metabolism 3.5 years postpartum. Multivariable linear and log-binomial regression with effects presented as mean increments (Δ) or relative risks (RRs) with 95% confidence intervals (CIs) were used to examine the association between late-pregnancy dysglycemia and outcomes. Linear mixed-effects models were used to study the longitudinal development of offspring body mass index (BMI) *z-*scores. The contribution of late-pregnancy dysglycemia to the association between maternal pre-conception obesity and offspring BMI was estimated using mediation analysis. In all, 898 mother–child pairs were included in this unplanned interim analysis. Among obese mothers with negative testing for GDM (*n =* 448), those with late-pregnancy dysglycemia (*n =* 135, 30.1%) had higher proportions of excessive total gestational weight gain (GWG), excessive third-trimester GWG, and offspring with large-for-gestational-age birth weight than those without. Besides higher birth weight (Δ 192 g, 95% CI 100–284) and cord-blood C-peptide concentration (Δ 0.10 ng/ml, 95% CI 0.02–0.17), offspring of these women had greater weight gain during early childhood (Δ BMI *z-*score per year 0.18, 95% CI 0.06–0.30, *n =* 262) and higher BMI *z-*score at 4 years (Δ 0.58, 95% CI 0.18–0.99, *n =* 43) than offspring of the obese, GDM-negative mothers with normal HbA_1c_ values at delivery. Late-pregnancy dysglycemia in GDM-negative mothers accounted for about one-quarter of the association of maternal obesity with offspring BMI at age 4 years (*n =* 151). In contrast, childhood BMI *z-*scores were not affected by a diagnosis of GDM in obese pregnancies (GDM-positive: 0.58, 95% CI 0.36–0.79, versus GDM-negative: 0.62, 95% CI 0.44–0.79). One mechanism triggering late-pregnancy dysglycemia in obese, GDM-negative mothers was related to excessive third-trimester weight gain (RR 1.72, 95% CI 1.12–2.65). Furthermore, in the maternal population, we found a 4-fold (RR 4.01, 95% CI 1.97–8.17) increased risk of future prediabetes or diabetes if obese, GDM-negative women had a high versus normal HbA_1c_ at delivery (absolute risk: 43.2% versus 10.5%). There is a potential for misclassification bias as the predominantly used GDM test procedure changed over the enrollment period. Further studies are required to validate the findings and elucidate the possible third-trimester factors contributing to future mother–child health status.

**Conclusions:**

Findings from this interim analysis suggest that offspring of obese mothers treated because of a diagnosis of GDM appeared to have a better BMI outcome in childhood than those of obese mothers who—following negative GDM testing—remained untreated in the last trimester and developed dysglycemia. Late-pregnancy dysglycemia related to uncontrolled weight gain may contribute to the development of child overweight and maternal diabetes. Our data suggest that negative GDM testing in obese pregnancies is not an “all-clear signal” and should not lead to reduced attention and risk awareness of physicians and obese women. Effective strategies are needed to maintain third-trimester glycemic and weight gain control among otherwise healthy obese pregnant women.

## Abstract 2

Regina Ensenauer and colleagues study the relationship between perinatal dysglycemia in obese mothers and later BMI in their children.

## Author summary

**Why was this study done?:**

- Pre-conception obesity is associated with an increased risk of pregnancy complications and adverse long-term health outcomes for the mother and her child.
- Obese pregnant women can develop impairments in glucose metabolism in late pregnancy despite prior negative testing for gestational diabetes mellitus (GDM).
- Yet, to date, guidelines on obesity in pregnancy and GDM have focused only on early glucose screening rather than targeting factors relevant to the last trimester of pregnancy.
- To evaluate whether recommendations on management of obese pregnancies require optimization, additional evidence is needed on the consequences of late-pregnancy dysglycemia for long-term childhood and maternal outcomes.

**What did the researchers do and find?:**

- We performed an interim analysis of 898 obese and normal weight mothers and their offspring from the Programming of Enhanced Adiposity Risk in Childhood–Early Screening (PEACHES) cohort study (total *n =* 1,671) that recruited pregnant women in Germany from 2010 to 2015.
- Late-pregnancy dysglycemia predisposed the offspring of obese, GDM-negative mothers to higher weight gain in early childhood and a higher body mass index at age 4 years.
- Children of obese mothers treated because of a diagnosis of GDM appeared to have a better weight outcome than those of obese mothers who remained untreated following a negative GDM test and developed late-pregnancy dysglycemia.
- Obese, GDM-negative women with late-pregnancy dysglycemia also had a 4-fold higher risk of prediabetes or diabetes several years after delivery compared to those with normal glucometabolic status in late pregnancy.

**What do these findings mean?:**

- We suggest that a negative GDM test at the end of the second trimester should not be understood as an “all-clear signal” and should not result in reduced attention of caregivers and a false sense of security in the mothers.
- Guidelines to manage and maintain third-trimester glycemic and weight gain control are needed for “high risk” obese women.
- Further analyses and studies should validate the findings and investigate the possible role of third-trimester factors for future mother–child health.

## Introduction

Since up to two-thirds of women of reproductive age are now overweight or obese in European countries and the US [1,2], obesity in pregnancy and its consequences represent a major public health challenge [3]. In Germany, the prevalence of overweight and obesity is 38.1% (obesity: 15.4%) among women of childbearing age [4] and 35.8% (obesity: 14.2%) among pregnant women [5]. Obese women are 3 to 5.5 times more likely to develop gestational diabetes mellitus (GDM) than normal weight women [6], leading to an approximately 3- to 10-fold increased risk of developing type 2 diabetes mellitus (T2DM) later in life [7,8]. In addition, in offspring of obese women, the risk of adverse health outcomes such as the development of adiposity, T2DM, cardiovascular disease, and asthma is higher [9,10].

Despite maternal oral glucose tolerance test (OGTT) values within the reference range during pregnancy, children of mothers with pre-conception obesity are reported to have a higher rate of overweight [11]. Evidence from recent systematic reviews and meta-analyses even suggests a greater contribution to child overweight from maternal pre-conception obesity than from GDM [12,13]. Apart from genetic background and lifestyle factors related to maternal obesity, prenatal metabolic influences of an adipogenic intrauterine milieu seem to play a relevant role, as evident from higher rates of increased offspring body fat at birth [14,15]. Potentially modifiable factors in the relationship between maternal gestational obesity and offspring childhood overweight may be linked to mechanisms of intrauterine lipotoxicity, including inflammatory changes and oxidative stress, and/or glucometabolic alterations that could exert effects during sensitive gestational periods.

We previously found high glycated hemoglobin (HbA_1c_) levels (≥5.7% [39 mmol/mol]) at delivery in about one-third of obese pregnant women [16,17], despite negative testing for GDM according to the International Association of Diabetes and Pregnancy Study Groups (IADPSG) criteria at the end of the second trimester [18]. This finding suggested the presence of relevant dysglycemia in late pregnancy, which was in turn associated with persistently abnormal glucose metabolism in the obese women postpartum and a higher rate of macrosomia in their offspring at birth [17]. However, assessing dysglycemia in the last trimester of pregnancy is not part of routine healthcare for obese women to date, and available guidelines on obesity in pregnancy have focused only on early glucose screening rather than addressing factors pertinent to the last third of pregnancy [19,20].

Therefore, in order to evaluate whether recommendations on gestational management of otherwise healthy obese women need to be optimized, further evidence is required as to whether such dysglycemia in late pregnancy could represent a long-term risk for offspring developing overweight later in childhood. In the prospective Programming of Enhanced Adiposity Risk in Childhood–Early Screening (PEACHES) cohort study, we had a unique set of longitudinal data on “high risk” obese mothers and their children including trimester-specific data on glucose metabolism that allowed us to address this question. Such clarification is of particular relevance to designing efficacious intervention and prevention strategies in the susceptible time window of late pregnancy.

## Methods

### Study design and participants

PEACHES is an ongoing prospective mother–child cohort study (*n =* 1,671) on pregnant women recruited between August 2010 and December 2015 during their first contact at maternity clinics (4–6 weeks before due date) in 23 departments of obstetrics and gynecology in the Munich area, Bavaria (southern Germany); the University Hospital of Düsseldorf (western Germany); and parts of northern Germany. In brief, the long-term effect of pre-conception maternal obesity on the development of overweight and associated metabolic diseases in both mothers and their offspring is being assessed, as described elsewhere [16,17]. The entire cohort comprises pre-conceptionally obese (body mass index [BMI] ≥ 30 kg/m^2^) or normal weight (BMI 18.5–24.9 kg/m^2^) women without preexisting diseases including type 1 diabetes mellitus (T1DM) or T2DM. The study was approved by the local ethics committee of the Ludwig-Maximilians-Universität München, Germany (protocol no. 165–10). Written informed consent was obtained from all participants. The study protocol is provided as S1 Study Protocol. This study is reported as per the Strengthening the Reporting of Observational Studies in Epidemiology (STROBE) guidelines (S1 STROBE Checklist). Data for this unplanned interim analysis were retrieved from the PEACHES database in August 2017.

### Procedures

#### Inclusion criteria for analysis

We included women in the data analysis if they were pre-conceptionally obese, were white, had a singleton live birth, and had not been diagnosed with T1DM or T2DM. Normal weight women who tested negative for GDM and had normal levels of HbA_1c_ at delivery were also eligible to be included in the analysis. Women with incomplete data on pre-conception BMI, GDM status, maternal HbA_1c_ at delivery, or confounding variables were excluded from the analysis.

#### Exposure variables

Obese and normal weight women who met the inclusion criteria for the analysis had GDM testing (50-g glucose challenge test [GCT] or 75-g OGTT) between 12 weeks and 1 day and 32 weeks and 6 days of gestation (median 25 weeks and 3 days, interquartile range [IQR] 3 weeks and 4 days). We included women with a negative GDM test performed before 23 weeks and 1 day [21] if absence of GDM was confirmed later in pregnancy (according to the IADPSG recommendation [18]). Blood glucose concentrations were obtained either from the pregnancy record booklet (“Mutterpass”) issued to every pregnant woman at her first antenatal visit in Germany or requested directly from the gynecologist. The pregnancy record booklet contains comprehensive information on ultrasound checkups, laboratory assessments, and weight measurements at multiple times collected by the gynecologist during antenatal care visits. GDM testing was defined as negative when none, and positive when 1 or more, of the 3 glucose concentrations of a 75-g OGTT met or exceeded the reference values according to the IADPSG criteria (1-step procedure): fasting glucose ≥ 5.1 mmol/l, 1-h post-load glucose ≥ 10 mmol/l, or 2-h post-load glucose ≥ 8.5 mmol/l [18]. In the 2-step procedure, a positive 50-g GCT, defined as 1-h post-load glucose concentration ≥ 7.8 mmol/l [22], was followed by a 75-g OGTT according to IADPSG diagnostic criteria [18]. In contrast to women with a negative test result (GDM-negative), those diagnosed with GDM (GDM-positive) received recommendations on treatment with insulin and/or diet, obtained advice on weight gain goals, and were monitored until the end of pregnancy.

Maternal HbA_1c_ concentration at delivery was measured in venous blood, following prompt transportation to a central laboratory, using high performance liquid chromatography (HPLC) via cation-exchange chromatography with a Tosoh G8 HPLC Analyzer (Tosoh Bioscience, Stuttgart, Germany) (interassay coefficient of variation ≤ 2.0%, analytic bias ≤ 0.05% HbA_1c_ at a target value of 5.33% [35 mmol/mol]). We used the term “late-pregnancy dysglycemia” when the maternal HbA_1c_ value at delivery was greater than or equal to the cut-off 5.7% (39 mmol/mol), as defined previously [17]. Information on the women’s iron supplementation and their red blood cell indices were used to exclude iron deficiency anemia as a potential cause of HbA_1c_ elevation [23].

#### Outcome variables: Offspring weight and metabolic outcomes

Short-term offspring outcomes included both absolute birth weight and large-for-gestational-age (LGA) birth weight, defined as >90th percentile [24], and were extracted from birth records. Cord blood was centrifuged (2,500*g*, 22°C) and sent to a central laboratory for analysis of C-peptide by a chemiluminescence immunoassay (Architect i2000, Abbott Wiesbaden, Germany). C-peptide values were dichotomized based on the 90th-percentile cutoff of the distribution among offspring from the normal weight, healthy (GDM-negative and HbA_1c_ < 5.7% at delivery) mothers in the PEACHES cohort (≥0.94 ng/ml [0.31 nmol/l]). Long-term outcomes in children included offspring’s BMI *z-*scores at 2, 3, and 4 years. Anthropometric data were obtained from records of the regular well-child care visits conducted by trained professionals of the preventive health program offered to all children in Germany. Age- and sex-specific BMI *z-*scores were calculated according to World Health Organization (WHO) Child Growth Standards [25].

#### Outcome variables: Maternal postpartum follow-up

At a follow-up visit several years postpartum, to which women were consecutively invited for evaluation of their metabolic health and body composition [17], maternal HbA_1c_ and glucose concentrations of an OGTT were measured. Postpartum maternal body weight and height were determined using a digital scale (Clara 803, Seca, Hamburg, Germany) with an accuracy of 0.1 kg and a stadiometer (model 213, Seca) with an accuracy of 0.1 cm. Body fat mass was determined by bioelectrical impedance analysis (BIA) (body composition analyzer BC-420 MA, Tanita, Sindelfingen, Germany). Waist and hip circumferences were measured to the nearest 0.1 cm using standardized protocols as recommended by WHO [26], and waist-to-hip ratio was calculated. Each anthropometric measurement was carried out 3 times consecutively by the same trained investigator and averaged for analysis.

Follow-up maternal HbA_1c_ was measured in EDTA plasma by HPLC via cation-exchange chromatography with a Variant II Turbo (BioRad, Hercules, California, US). Analysis of serum glucose concentrations was performed using the hexokinase method on an AU 5800 analyzer (Beckman Coulter, Krefeld, Germany). The presence of T2DM (fasting glucose ≥ 7.0 mmol/l or 2-h post-load glucose of a 75-g OGTT ≥ 11.1 mmol/l or HbA_1c_ ≥ 6.5% [48 mmol/mol]) and prediabetes (fasting glucose 5.6 mmol/l to 6.9 mmol/l or 2-h post-load glucose of a 75-g OGTT 7.8 mmol/l to 11.0 mmol/l or HbA_1c_ 5.7% to 6.4% [39 to 47 mmol/mol]) was determined [22].

#### Confounders

We extracted information on maternal age at conception and weight measurements from the mothers’ pregnancy record booklets. Pre-conception BMI was based on data measured at the first antenatal visit if the visit was before 12 weeks and 6 days of gestation (92.5% of participants). When the first antenatal visit was later than the 13th week of gestation (7.5% of participants), pre-conception weight was used as reported by the woman at this first visit and documented in the pregnancy record booklet. Pre-conception BMI groups were defined according to WHO categories [27].

Total gestational weight gain (GWG) in pregnancy was defined as the difference between the last measured weight before delivery and pre-conception weight and classified as inadequate, adequate, or excessive according to the BMI-specific recommendations of the Institute of Medicine [28]. Third-trimester GWG (i.e., between 27 weeks of gestation and delivery) was calculated using the difference between the first (mean 28 weeks and 3 days [standard deviation (SD) 1 week and 2 days]) and last (mean 38 weeks and 4 days [SD 2 weeks and 2 days]) documented weight in the third trimester. To categorize third-trimester GWG as excessive or non-excessive for each woman, we calculated the average third-trimester weight gain per week (third-trimester GWG divided by weeks between the 2 weight measurements) [29] and compared it to the respective BMI-specific recommendations for weight gain per week of the Institute of Medicine [28].

Offspring sex and gestational age were extracted from birth records. Information on breastfeeding and treatment for GDM was collected using a questionnaire sent to each participant. Breastfeeding data were dichotomized as “≥1 month exclusively without interruption” or “never or <1 month exclusively.” Data on smoking and iron supplementation during pregnancy were obtained twice, via the questionnaire and via a standardized telephone interview shortly after delivery. Reported smoking at either assessment was categorized as maternal smoking at “any time during pregnancy” (versus “no time during pregnancy”).

### Statistical analysis

Aspects of the analysis plan were written prior to the analysis (S1 Study Protocol). However, there was no detailed prospective plan for the current interim analysis. The confounder adjustment and modeling strategy were modified in response to reviewers’ suggestions. This unplanned analysis was triggered to present interim results that could potentially provide guidance on how to proceed with the future research of the cohort.

We compared gestational, offspring, and maternal characteristics by GDM status (negative or positive) and maternal HbA_1c_ level at delivery (high or normal) using Student’s *t* test or 1-way analysis of variance (ANOVA) and χ^2^ test as appropriate. After confirmation of a linear relationship between maternal HbA_1c_ at delivery and offspring BMI *z-*score at 4 years using B-splines [30], we performed linear regression to estimate the association between maternal HbA_1c_ at delivery (continuous) and offspring BMI *z-*score at 4 years.

The association of late-pregnancy dysglycemia with short- and long-term offspring and maternal outcomes among obese, GDM-negative mothers was examined using linear (continuous outcomes) and log-binomial (binary outcomes) regression; comparisons were relative to (i) obese, GDM-negative mothers with normal HbA_1c_ levels and (ii) obese, GDM-positive mothers. Effects are expressed as mean increments (Δ, linear regression) and relative risks (RRs, log-binomial regression) with 95% confidence intervals (CIs). In addition, the risk of developing late-pregnancy dysglycemia due to excessive third-trimester GWG was assessed in the group of obese, GDM-negative mothers using log-binomial regression. Models were adjusted for potential confounders including maternal pre-conception BMI, total GWG, maternal smoking at any time during pregnancy, and sex of the child; models for long-term childhood outcomes were additionally adjusted for exclusive breastfeeding ≥1 month. Potential confounders were chosen based on their demonstrated relationship to offspring childhood overweight [31]. The model for the maternal outcome prediabetes/T2DM was adjusted for maternal body fat percentage at 3.5 years postpartum.

To assess the longitudinal association of late-pregnancy dysglycemia (relative to the absence of late-pregnancy dysglycemia) with offspring BMI *z-*scores at ages 2, 3, and 4 years, we constructed linear mixed-effects models with random effects for intercept and time. Polynomial contrasts and interaction with group were tested to examine differential nonlinear time courses between both groups; the corresponding likelihood ratio test did not give strong evidence for a nonlinear time effect. Models were fitted using the R package “lme4” [32]. Missing data relate to the timing of recruitment into our cohort (i.e., offspring of mothers recruited after 2013 were too young to have their 4-year follow-up by August 2017) rather than loss to follow-up or withdrawal from participation. Thus, we assumed missingness at random for the follow-up data, which does not bias results of linear mixed-effects models.

Mediation analysis was conducted to assess whether late-pregnancy dysglycemia (as indicated by a high maternal HbA_1c_ at delivery) contributed to the association of maternal obesity with offspring BMI *z-*score in GDM-negative women [33]. First, we assessed the total effect of maternal pre-conceptional obesity on 4-year BMI *z-*score by comparing offspring of obese, GDM-negative women versus offspring of normal weight, GDM-negative women and adjusting for confounders as above. Subsequently, we adjusted for maternal HbA_1c_ at delivery (high versus normal) to estimate the direct effect of maternal pre-conceptional obesity on offspring 4-year BMI *z-*score. The difference between the total and direct effect provides a quantification of the potential contribution of late-pregnancy dysglycemia to increased 4-year BMI *z-*score. These analyses were conducted in all obese and normal weight women for whom information on offspring 4-year BMI *z-*score was available.

The sample size for the analysis is compatible with the sample size calculation provided in the original protocol as presented in S1 Study Protocol. Therefore, the data analyzed provide sufficient power to detect relevant effects.

We formally considered a *p*-value < 0.05 to be statistically significant, ignoring possible alpha inflation. The statistical analysis was carried out with the statistical software package R version 3.3.1 [34].

## Results

### Study population

**Fig 1:**
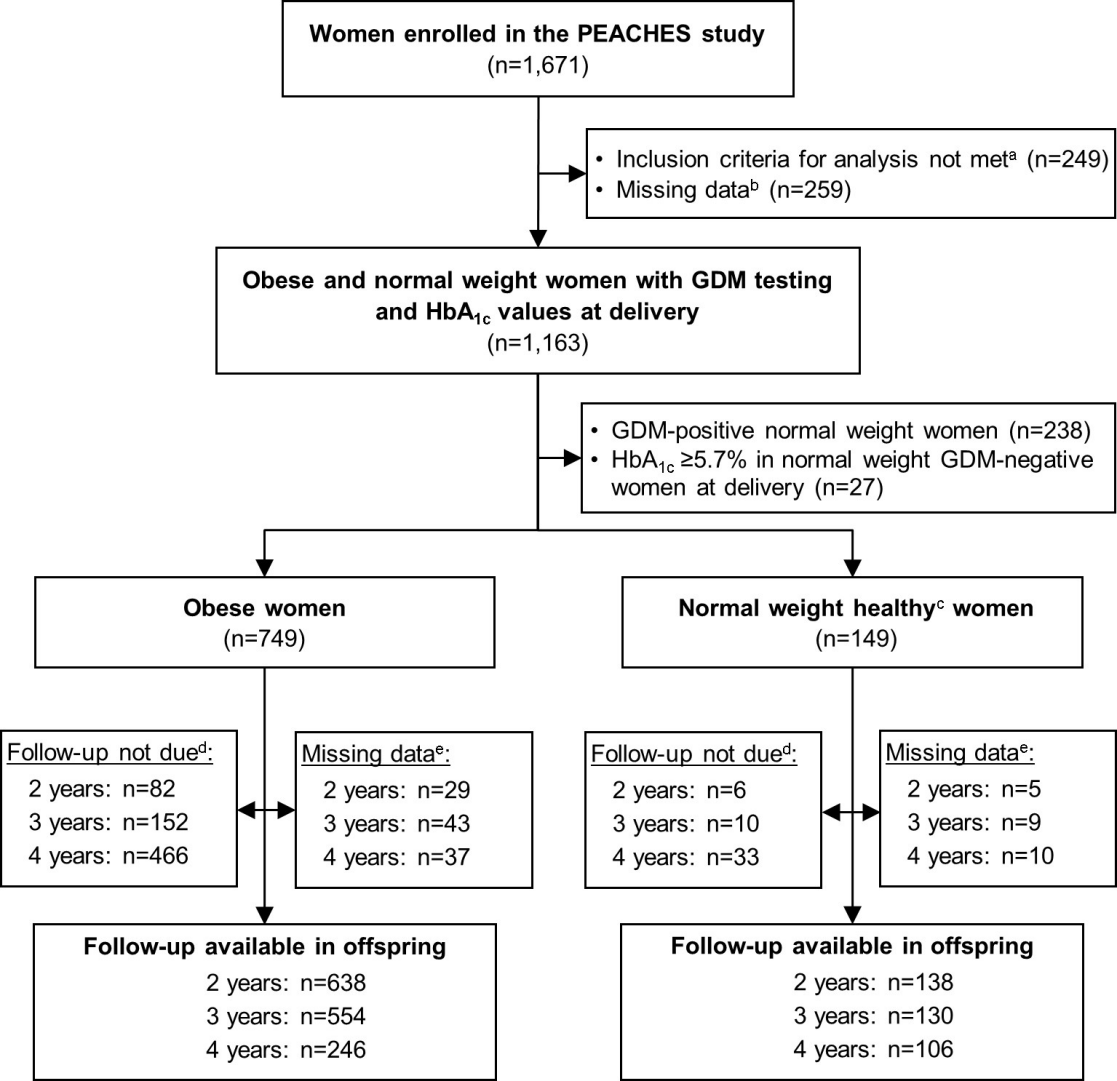
PEACHES study population and follow-up investigations of children. ^a^Did not meet inclusion criteria for analysis including pre-conception obesity or normal weight, singleton live birth, and absence of type 1 diabetes and type 2 diabetes. ^b^Missing information for at least 1 of the following variables: pre-conception body mass index group (normal weight or obese), GDM status (GDM-negative or GDM-positive), maternal HbA_1c_ at delivery (<5.7% [39 mmol/mol] or ≥5.7%), or confounding variables. ^c^“Healthy” defined as GDM-negative and HbA_1c_ < 5.7% at delivery. ^d^Offspring too young at the time of data retrieval from the PEACHES database. ^e^Loss to follow-up or withdrawal from participation. GDM, gestational diabetes mellitus; HbA_1c_, glycated hemoglobin; PEACHES, Programming of Enhanced Adiposity Risk in Childhood–Early Screening.

A total of 898 women (749 obese and 149 normal weight) of the PEACHES cohort were eligible to be included in our analyses (Fig 1). Compared with women excluded from analysis due to missing data (*n =* 259), the included women were more likely to have a negative GDM test and had higher total GWG (S1 Table). Table 1 summarizes maternal and offspring characteristics of the study sample by GDM status and the presence or absence of late-pregnancy dysglycemia as indicated by a high or normal maternal HbA_1c_ level at delivery, respectively. More than one-third of obese women had a high HbA_1c_ value at delivery. In the subgroup of obese, GDM-negative women, 30% had a high HbA_1c_ value at delivery, whereas this proportion was 45% in the group of obese, GDM-positive women, who received various treatment regimens for their condition.

**Table 1:** Characteristics of the PEACHES study population: data on main exposure, maternal and offspring outcomes, and potential confounders.

Maternal/child characteristic	Normal weight mothers, GDM−, normal HbA_1c_	Obese mothers stratified by glucometabolic status during pregnancy (GDM testing) and at delivery (HbA_1c_)			
		GDM−, normal HbA_1c_	GDM−, high HbA_1c_	GDM+, normal HbA_1c_	GDM+, high HbA_1c_
**Maternal characteristics during pregnancy**					
*N*	149	313	135	165	136
Age at conception, years	32.9 (4.8)	30.8 (5.1)	31.2 (5.9)	**32.6 (4.9)**	**32.8 (5.0)**
Pre-conception BMI, kg/m^2^	21.9 (1.6)	36.2 (5.1)	36.8 (5.4)	36.3 (5.2)	**38.5 (5.5)**
Fasting glucose at GDM testing, mmol/l^a^	4.36 (0.44)	4.41 (0.40)	**4.52 (0.33)**	**5.30 (0.71)**	**5.65 (0.89)**
Smoking at any time during pregnancy	20 (13.4%)	84 (26.8%)	34 (25.2%)	42 (25.5%)	47 (34.6%)
GDM treatment					
Diet only	0 (0.0%)	0 (0.0%)	0 (0.0%)	**90 (54.6%)**	**63 (46.3%)**
Insulin and diet	0 (0.0%)	0 (0.0%)	0 (0.0%)	**54 (32.7%)**	**38 (27.9%)**
Insulin only	0 (0.0%)	0 (0.0%)	0 (0.0%)	**21 (12.7%)**	**35 (25.8%)**
**Maternal characteristics at delivery**					
Excessive total GWG	51 (34.2%)	209 (66.8%)	**104 (77.0%)**	**93 (56.4%)**	**75 (55.1%)**
Total GWG, kg	14.8 (4.9)	12.7 (7.5)	**14.9 (7.6)**	**9.8 (7.7)**	**10.8 (7.8)**
Excessive third-trimester GWG	75 (50.3%)	233 (74.4%)	**115 (85.8%)**	**78 (47.3%)**	**90 (66.7%)**
Third-trimester GWG, kg	5.0 (2.5)	5.2 (3.4)	**6**.**0 (3**.**5)**	**2**.**8 (3**.**6)**	**4**.**2 (3**.**8)**
HbA_1c_ at delivery, percent^b^	5.3 (0.2)	5.3 (0.2)	**5.9 (0.2)**	5.3 (0.3)	**6.0 (0.4)**
**Child characteristics at birth**					
Sex: female	77 (51.7%)	148 (47.3%)	60 (44.4%)	85 (51.5%)	65 (47.8%)
Gestational age, weeks	40.4 (1.2)	39.9 (1.5)	40.0 (1.4)	39.7 (1.3)	**39.5 (1.4)**
Birth weight, g	3,456 (438)	3,454 (450)	**3,676 (480)**	3,440 (468)	**3,569 (484)**
Birth weight: LGA	8 (5.4%)	21 (6.7%)	**23 (17.0%)**	15 (9.1%)	**24 (17.7%)**
Cord-blood C-peptide, ng/ml^c^	0.48 (0.36)	0.51 (0.34)	**0**.**60 (0**.**40)**	0.57 (0.41)	**0**.**63 (0**.**49)**
Breastfeeding (exclusive), ≥1 month	117 (78.5%)	166 (53.0%)	67 (49.6%)	83 (50.3%)	59 (43.4%)
**Maternal characteristics postpartum**					
*N*	47	86	37	52	46
Time after index pregnancy, years	4.4 (0.7)	3.9 (0.9)	**3.1 (0.9)**	3.6 (0.9)	**3.4 (0.8)**
BMI, kg/m^2^	22.8 (1.9)	38.8 (6.7)	39.4 (6.8)	38.1 (5.6)	40.5 (7.3)
Waist-to-hip ratio	0.80 (0.05)	0.84 (0.06)	**0.87 (0.06)**	**0.87 (0.05)**	**0.88 (0.07)**
Percentage body fat by BIA, percent	29.5 (5.3)	46.3 (4.8)	46.4 (4.6)	45.4 (3.9)	46.6 (5.2)
**Child age (months) at follow-up**					
At 2-year follow-up	24.5 (1.3)	24.2 (1.1)	24.3 (1.2)	24.1 (1.3)	24.3 (1.2)
At 3-year follow-up	36.9 (1.1)	36.6 (1.0)	36.6 (1.0)	36.6 (1.3)	36.8 (1.3)
At 4-year follow-up	48.7 (1.5)	48.8 (1.0)	49.0 (1.3)	49.0 (1.0)	48.7 (0.9)

Data are mean (SD) or *n* (%) and tested with regard to the obese, GDM−, normal HbA_1c_ group using Student’s *t* test for continuous and χ^2^ test for categorical variables. High HbA_1c_ is HbA_1c_ ≥ 5.7% (39 mmol/mol)]; normal HbA_1c_ is HbA_1c_ < 5.7%. Bold font indicates *p* < 0.05. Participants with any missing information for baseline characteristics were excluded.

^a^GDM testing was performed at median 25 weeks and 3 days of gestation (interquartile range 3 weeks and 4 days). To convert glucose mmol/l to mg/dl, multiply by 18.018.

^b^To convert HbA_1c_ percent to mmol/mol: IFCC HbA_1c_ unit (mmol/mol) = [10.93 × DCCT/NGSP unit (%)] − 23.50.

^c^To convert C-peptide ng/ml to nmol/l, multiply by 0.331.

BIA, bioelectrical impedance analysis; BMI, body mass index; DCCT/NGSP, Diabetes Control and Complications Trial/National Glycohemoglobin Standardization Program; GDM, gestational diabetes mellitus; GWG, gestational weight gain; HbA_1c_, glycated hemoglobin; IFCC, International Federation of Clinical Chemistry and Laboratory Medicine; LGA, large-for-gestational-age; PEACHES, Programming of Enhanced Adiposity Risk in Childhood–Early Screening; SD, standard deviation.

Testing for GDM was done using the 1-step procedure in 64% (*n =* 571) of obese and normal weight mothers, while 36% (*n =* 327) underwent the 2-step procedure. In the latter group, 66 had a positive GCT (1-h post-load glucose, median 10.2 mmol/l [IQR 2.5]), and 261 had a negative GCT (1-h post-load glucose, median 6.0 mmol/l [IQR 1.7]). A higher proportion of obese mothers diagnosed as GDM-negative underwent a 1-step compared to a 2-step GDM procedure (55.4%, 95% CI 50.8%–60.0%, versus 44.6%, 95% CI 40.0%–49.2%). However, despite the differences in the GDM test procedure used among these women, the proportion of obese, GDM-negative women who developed dysglycemia in late pregnancy was similar (1-step versus 2-step: 29.8%, 95% CI 24.1%–35.6%, versus 30.5%, 95% CI 24.1%–36.9%).

There was no noticeable difference in hemoglobin levels and red blood cell indices between obese, GDM-negative women with high compared to normal levels of HbA_1c_ at delivery (mean hemoglobin: 12.0 g/dl, 95% CI 11.8–12.2, versus 12.2 g/dl, 95% CI 12.0–12.4, and mean corpuscular hemoglobin: 28.6 pg, 95% CI 27.9–29.2, versus 28.9 pg, 95% CI 28.7–29.2). Those with high HbA_1c_ values at delivery had higher 75-g OGTT glucose concentrations, albeit below diagnostic cutoffs, at the time of GDM testing in pregnancy than those with normal HbA_1c_ values at delivery (S2 Table). Further, obese, GDM-negative women with a high HbA_1c_ at delivery had higher mean total and third-trimester GWG (Table 1). They were also more likely to have newborns with LGA birth weights, comparable to the proportion seen in obese, GDM-positive women with high HbA_1c_ values at delivery, and higher cord-blood C-peptide concentrations. In contrast to these women, obese, GDM-positive women had lower mean total and third-trimester GWG, irrespective of their HbA_1c_ level at delivery (Table 1), potentially due to risk awareness, treatment of GDM with insulin and/or diet, and tight supervised control.

### Prenatal risk factors for increased childhood weight status

**Fig 2:**
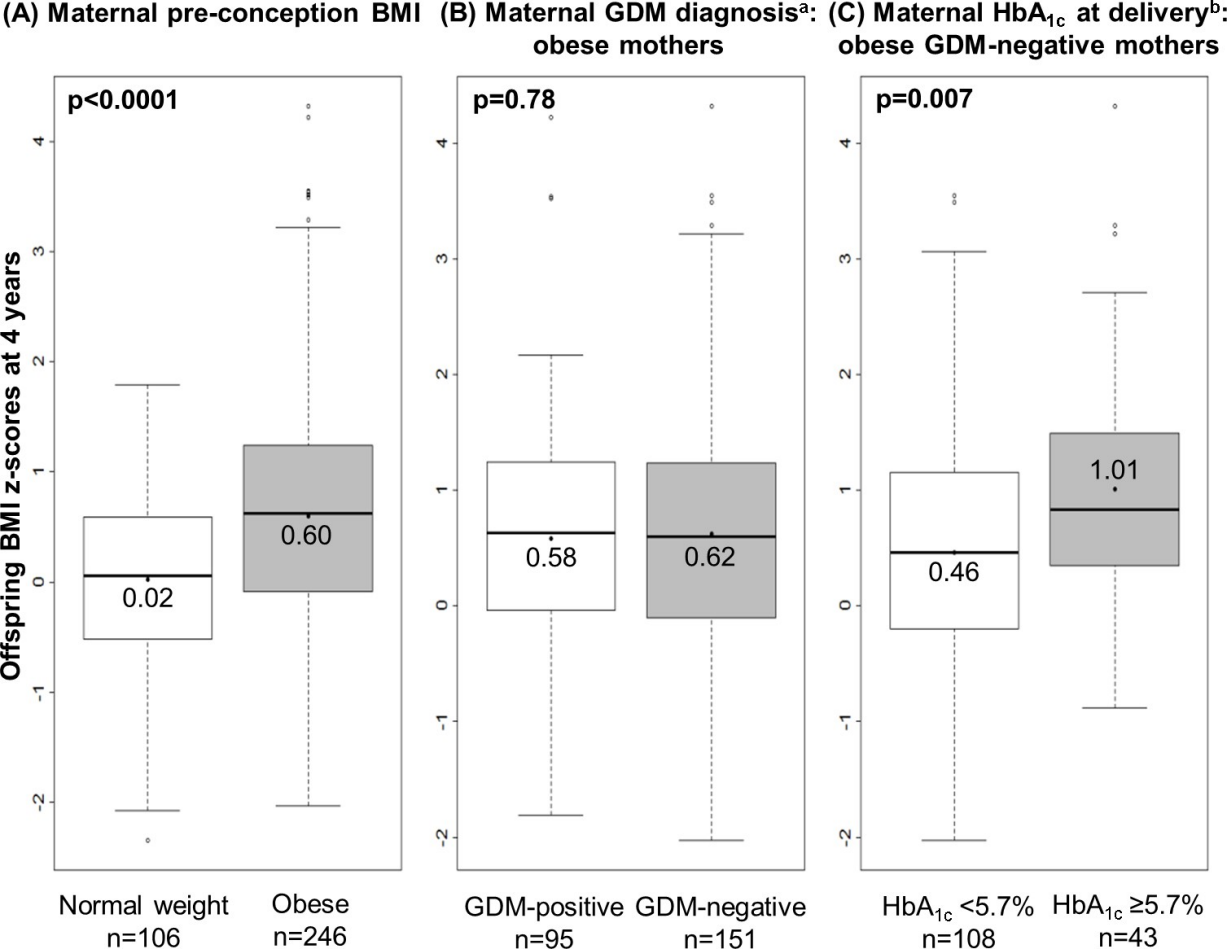
Offspring 4-year BMI *z-*score by maternal pre-conception weight status and glucometabolic status in pregnancy and at delivery. Stratification of maternal groups was performed in enrolled mother–child pairs with offspring 4-year BMI *z-*scores according to the (A) pre-conception BMI group of 352 mothers, (B) positive or negative testing for GDM in 246 obese women, and (C) HbA_1c_ at delivery in 151 obese, GDM-negative women. Data are shown as median (horizontal lines within the boxes), 25th and 75th centile (lower and upper boundaries of the boxes), 1.5 times the interquartile range (whisker ends), and outliers (circles). Numerical values and dots within the boxes represent unadjusted mean 4-year BMI *z-*score of offspring. Differences between groups were tested using Student’s *t* test. ^a^According to the International Association of Diabetes and Pregnancy Study Groups criteria [18]. ^b^Dichotomized based on a predefined cutoff value of ≥5.7% (39 mmol/mol) [17]. BMI, body mass index; GDM, gestational diabetes mellitus; HbA_1c_, glycated hemoglobin.

Offspring weight status was studied in children of obese and normal weight mothers until age 4 years (Fig 1). The follow-up rate in children was 88% at 4 years (S3 Table) without relevant differences in characteristics between those with and those without follow-up (S4 Table). Fig 2 shows the relation of prenatal risk factors with offspring BMI *z-*score at age 4 years. As expected, children of women with pre-conception obesity had a higher mean 4-year BMI *z-*score (0.60, 95% CI 0.46–0.74, versus 0.02, 95% CI −0.14 to 0.18) than children of normal weight women (Fig 2A). Surprisingly, further stratification by GDM status among obese mothers did not show any noticeable differences in offspring mean 4-year BMI *z-*score between the 2 strata (0.62, 95% CI 0.44–0.79, versus 0.58, 95% CI 0.36–0.79) (Fig 2B). However, offspring of obese, GDM-negative women with high HbA_1c_ at delivery had a higher mean 4-year BMI *z-*score than offspring of obese, GDM-negative women with normal HbA_1c_ at delivery (1.01, 95% CI 0.68–1.35, versus 0.46, 95% CI 0.24–0.67) (Fig 2C). An analysis using HbA_1c_ as a continuous variable showed that among offspring of obese, GDM-negative mothers, the child’s BMI *z-*score at 4 years increased by 0.07 (95% CI 0.01–0.12) for every 0.1% increase in maternal HbA_1c_ at delivery (S1 Fig).

### Late-pregnancy dysglycemia and longitudinal offspring outcomes in early childhood

Offspring of obese, GDM-negative mothers with late-pregnancy dysglycemia had higher mean birth weight and cord-blood C-peptide concentration than newborns of the respective mothers with normal HbA_1c_ at delivery (Table 2). High (≥0.94 ng/ml [0.31 nmol/l]) cord-blood C-peptide concentrations were associated with a 2-fold (RR 2.01, 95% CI 1.07–3.78) increase in the risk of LGA birth weight in these babies. At age 4 years, these children had higher mean BMI *z-*score than all other groups except for obese, GDM-positive women with high HbA_1c_ levels at delivery (S2 Fig). The slope of the BMI *z-*score curve between the ages of 2 and 4 years among children of obese, GDM-negative mothers with high HbA_1c_ at delivery was positive (0.08), while it was negative (−0.10) among offspring of the respective mothers with normal HbA_1c_ at delivery, for a net difference in slope of 0.18 (95% CI 0.06–0.30) (Table 2). In contrast, birth outcomes, offspring BMI *z-*score slope, and 4-year BMI *z-*score were not different between obese, GDM-positive mothers with high versus normal maternal HbA_1c_ levels at delivery (S5 Table).

**Table 2:** Late-pregnancy dysglycemia in obese, GDM-negative mothers and offspring outcomes.

Child outcome	Control group (obese, GDM−, normal HbA_1c_)		Maternal late-pregnancy dysglycemia (obese, GDM−, high HbA_1c_)	
	*N*	Mean (95% CI)	*N*	Mean increment Δ (95% CI) with respect to control group
At delivery^a^				
Birth weight, g	313	3,454 (3,404 to 3,504)	135	**192 (100 to 284)**
Cord-blood C-peptide, ng/ml^b^	296	0.51 (0.47 to 0.55)	130	**0.10 (0.02 to 0.17)**
Long-term follow-up^c^				
BMI *z-*score change per year^d^	595	−0.10 (−0.17 to −0.03)	262	**0.18 (0.06 to 0.30)**
BMI *z-*score at 4 years^e^	108	0.46 (0.24 to 0.68)	43	**0.58 (0.18 to 0.99)**

Mean increments in offspring outcomes by high maternal HbA_1c_ (≥5.7% [39 mmol/mol]) at delivery are shown relative to the obese, GDM−, normal HbA_1c_ group. Bold font indicates *p* < 0.05.

^a^Based on linear regression models, adjusted for maternal pre-conception BMI, total gestational weight gain, maternal smoking at any time during pregnancy, and sex of the child.

^b^To convert C-peptide ng/ml to nmol/l, multiply by 0.331.

^c^Adjusted for maternal pre-conception BMI, total gestational weight gain, maternal smoking at any time during pregnancy, and exclusive breastfeeding ≥1 month.

^d^Based on linear mixed-effects model.

^e^Based on linear regression model.

BMI, body mass index; CI, confidence interval; GDM, gestational diabetes mellitus; HbA_1c_, glycated hemoglobin.

**Fig 3:**
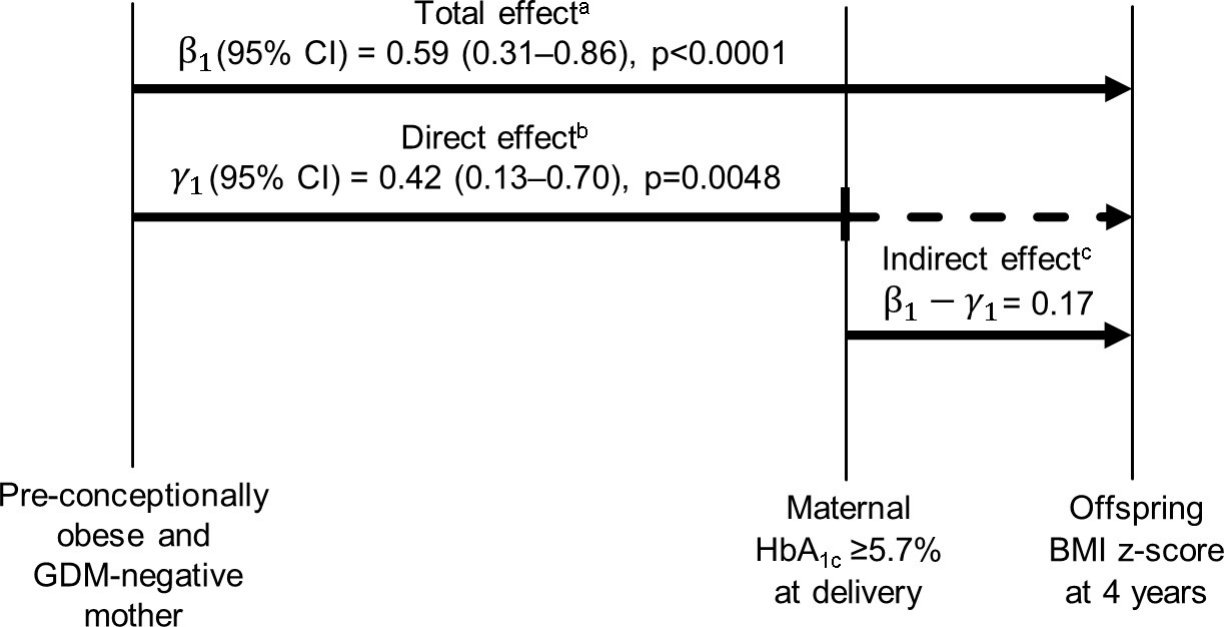
Contribution of late-pregnancy dysglycemia to the effect of maternal obesity on increased weight status in 4-year-old children. Mediation analysis was performed to study the total effect of pre-conception obesity in GDM-negative mothers on offspring BMI *z-*score at age 4 years, comprising the direct effect of maternal obesity and the indirect effect of late-pregnancy dysglycemia (as indicated by a high maternal HbA_1c_ [≥5.7%] at delivery). Data are coefficients derived from linear regression models, adjusted for maternal smoking at any time during pregnancy, total GWG, and exclusive breastfeeding ≥1 month. ^a^Estimated as β_1_ from: BMIz_4 years_ = β_0_ + β_1_ * maternal obesity_yes/no_ + β_2_ * maternal smoking_yes/no_ + β_3_ * total GWG + β_4_ * breastfeeding_yes/no_^b^Estimated as γ_1_ from: BMIz_4 years_ = γ_0_ + γ_1_ * maternal obesity_yes/no_ + γ_2_ * maternal smoking_yes/no_ + γ_3_ * total GWG + γ_4_ * breastfeeding_yes/no_ + γ_5_ * HbA_1c≥5.7% or <5.7%_. ^c^Calculated as β_1_−γ_1_ BMI, body mass index; GDM, gestational diabetes mellitus; GWG, gestational weight gain; HbA_1c_, glycated hemoglobin.

To quantify the contribution of late-pregnancy dysglycemia to increased BMI *z-*scores in children of obese, GDM-negative women, we conducted mediation analysis (Fig 3). Compared to offspring of normal weight women (*n =* 106), offspring of mothers with pre-conception obesity (*n =* 151) had 0.59 (95% CI 0.31–0.86) units higher BMI *z-*scores at age 4 years; following adjustment for HbA_1c_ at delivery (high versus normal), offspring of mothers with pre-conception obesity had 0.42 (95% CI 0.13–0.70) units higher BMI *z-*scores at 4 years. Consequently, the proportion of the effect of maternal obesity on offspring BMI *z-*score that is contributed by late-pregnancy dysglycemia was (0.59 − 0.42)/0.59 = 29%.

### Excessive weight gain and deterioration of glucometabolic control in the last trimester following negative GDM testing

Next, we investigated whether weight gain has a role in triggering dysglycemia in the last trimester. Children of obese mothers with GDM who received treatment and weight monitoring because of their diagnosis appeared to have better short- and long-term BMI outcomes than children of obese mothers who remained untreated following negative GDM testing and developed dysglycemia later on (Tables 2, S5 and 3). In offspring of obese, GDM-negative mothers (untreated) with late-pregnancy dysglycemia, the BMI *z-*score slope during the early childhood years was marginally increased (0.13, 95% CI −0.02 to 0.27) compared to that in offspring of obese, GDM-positive mothers (treated) (Table 3). We further found that obese, GDM-negative women with excessive GWG in the third trimester were more likely to have a high HbA_1c_ at delivery compared to those without excessive third-trimester GWG (RR 1.72, 95% CI 1.12–2.65).

**Table 3:** GDM status and late-pregnancy dysglycemia in obese mothers and offspring outcomes.

Child outcome	Control group (obese, GDM+, treated)		Obese, GDM−, high HbA_1c_ (untreated)	
	*N*	Mean (95% CI)	*N*	Mean increment Δ (95% CI) with respect to control group
At delivery^a^				
Birth weight, g	301	3,498 (3,444 to 3,552)	135	**134 (28 to 239)**
Cord-blood C-peptide, ng/ml^b^	286	0.60 (0.54 to 0.66)	130	−0.04 (−0.14 to 0.07)
Long-term follow-up^c^				
BMI *z-*score change per year^d^	576	−0.05 (−0.11 to 0.03)	262	0.13 (−0.02 to 0.27)
BMI *z-*score at 4 years^e^	95	0.58 (0.36 to 0.79)	43	**0.52 (0.07 to 0.97)**

Mean increments in offspring outcomes in obese, GDM−, high HbA_1c_ mothers are shown relative to the entire obese, GDM-positive group (regardless of HbA_1c_ level at delivery). Bold font indicates *p* < 0.05.

^a^Based on linear regression models, adjusted for maternal pre-conception BMI, total gestational weight gain, maternal smoking at any time during pregnancy, and sex of the child.

^b^To convert C-peptide ng/ml to nmol/l, multiply by 0.331.

^c^Adjusted for maternal pre-conception BMI, total gestational weight gain, maternal smoking at any time during pregnancy, and exclusive breastfeeding ≥1 month.

^d^Based on linear mixed-effects model.

^e^Based on linear regression model.

BMI, body mass index; CI, confidence interval; GDM, gestational diabetes mellitus; HbA_1c_, glycated hemoglobin.

### Late-pregnancy dysglycemia in obese, GDM-negative women and their future diabetes risk

The relevance of late-pregnancy glucometabolic control in obese, GDM-negative women for their own metabolic health years after delivery (median 3.5 years [IQR 1.2]) was substantiated by follow-up data of the maternal PEACHES population (Tables 4 and S6; *n =* 123). At 3.5 years postpartum, HbA_1c_ and fasting and 1-h post-load glucose concentrations in obese, GDM-negative women with late-pregnancy dysglycemia were elevated, contributing to a 4-fold (RR 4.01, 95% CI 1.97–8.17) increased risk of developing T2DM or prediabetes, as compared to those with normal HbA_1c_ levels at delivery (absolute risk: 43.2% versus 10.5%).

**Table 4:** Late-pregnancy dysglycemia in obese, GDM-negative mothers and glucose metabolism and T2DM/prediabetes risk 3.5 years postpartum^a^.

Maternal outcome 3.5 years postpartum	Control group (obese, GDM−, normal HbA_1c_)		Maternal late-pregnancy dysglycemia (obese, GDM−, high HbA_1c_)	
	*N*	Mean (95% CI)	*N*	Mean increment Δ (95% CI) with respect to control group
HbA_1c_, percent	86	5.19 (5.13 to 5.25)	37	**0.36 (0.25 to 0.46)**
Fasting glucose, mmol/l	86	4.77 (4.69 to 4.85)	37	**0.19 (0.05 to 0.33)**
1-h post-load glucose, mmol/l	84	7.06 (6.73 to 7.39)	37	**0.76 (0.13 to 1.38)**
2-h post-load glucose, mmol/l	84	5.68 (5.43 to 5.93)	37	0.21 (−0.28 to 0.69)
RR for T2DM/prediabetes^b^	86	1.00 (Ref.)	37	**4.01 (1.97 to 8.17)**

Mean increments in maternal postpartum parameters by high maternal HbA_1c_ (≥5.7% [39 mmol/mol]) at delivery are shown relative to the obese, GDM−, normal HbA_1c_ group. Data are based on linear regression models, adjusted for maternal body fat percentage 3.5 years postpartum. Bold font indicates *p* < 0.05.

^a^Maternal postpartum follow-up data not available in 325 obese, GDM-negative mothers due to loss to follow-up or withdrawal from participation (8.9%), consecutive pregnancy (10.8%), follow-up period too short (31.1%), or being currently in the time window for the follow-up visit (49.2%).

^b^Log-binomial regression model, adjusted for maternal body fat percentage 3.5 years postpartum. Presence of T2DM (fasting glucose ≥ 7.0 mmol/l or 2-h post-load glucose in 75-g OGTT ≥ 11.1 mmol/l or HbA_1c_ ≥ 6.5% [48 mmol/mol]) or prediabetes (fasting glucose 5.6 mmol/l to 6.9 mmol/l or 2-h post-load glucose in 75-g OGTT 7.8 mmol/l to 11.0 mmol/l or HbA_1c_ 5.7% to 6.4% [39 to 47 mmol/mol]) [22].

CI, confidence interval; GDM, gestational diabetes mellitus; HbA_1c_, glycated hemoglobin; OGTT, oral glucose tolerance test; Ref., reference; RR, relative risk; T2DM, type 2 diabetes mellitus.

## Discussion

Identifying crucial periods of developmental programming is important for designing effective interventions [14], considering that obesity and diabetes in pregnancy are the major transgenerational health burden to date [35,36]. As yet, to our knowledge, the occurrence of dysglycemia in the last trimester of pregnancy despite prior negative testing for GDM has not been considered a problem for long-term health outcomes of obese pregnancies and thus has not been included in respective clinical care guidelines as part of routine monitoring [19]. Our data suggest that late-pregnancy dysglycemia predisposes the offspring of obese, GDM-negative mothers to alterations in weight development during early childhood. Moreover, offspring of obese mothers treated and monitored because of a diagnosis of GDM appeared to have a better BMI outcome in childhood than those of obese mothers who—following negative GDM testing—remained untreated in the last trimester and developed an abnormal glucose metabolism.

Our hypothesis of a possible relevance of dysglycemia in the last trimester for longitudinal offspring outcomes was based on data from mothers with pregestational diabetes. Several studies in non-obese women with T1DM or T2DM have shown an association of elevated third-trimester HbA_1c_ with a 2- to 5-fold increased risk of LGA birth weight [37,38]. Even among the offspring of GDM-negative mothers in our obese cohort, a high HbA_1c_ at delivery was associated with a similarly increased risk of LGA birth weight (RR 2.03, 95% CI 1.17–3.53). The apparent “vulnerability” during this time window goes along with intense differentiation of the fetal pancreatic islet cells that are adaptive to glucose supply. The resulting hyperinsulinemia and increased offspring growth may lead to an insulin secretory defect, contributing to a lifelong higher risk of developing overweight and T2DM, as shown in animal studies [39]. However, information on the possible long-term impact of a dysglycemic intrauterine milieu in the last trimester on the next generation’s health in humans is scarce. Adult offspring of normal weight Danish mothers with T1DM and elevated blood glucose during late gestation presented with an increased risk of T2DM or prediabetes at age 22 years [40]. Earlier data from Pima Indians, among whom the prevalence of T2DM is extremely high, further suggest that offspring, as a result of their mothers’ third-trimester glucose tolerance, may develop adverse outcomes over time [41]: their risk of obesity was most pronounced by the age of 10 to 14 years, in addition to abnormal glucose metabolism emerging several years later. However, maternal pre-conception BMI was not considered. Our data from obese pregnancies with negative GDM testing suggest that disturbances in maternal glucose homeostasis in the last gestational weeks play a role in faster weight gain and early manifestation of an increased weight status in offspring at preschool age. Glucometabolic control in the last trimester seems critical for future BMI development, considering that children of obese mothers diagnosed with GDM and treated in the last trimester appear to have more favorable outcomes.

We found that an increased HbA_1c_ at delivery indeed reflects late-pregnancy dysglycemia based on our finding of higher glucose concentrations in the 75-g OGTT at GDM testing during pregnancy, albeit below diagnostic cutoffs, in obese, GDM-negative women with high versus normal HbA_1c_ values at delivery, similar to our previous findings [17]. The increased cord-blood C-peptide concentrations in the offspring of obese women with high HbA_1c_ values at delivery further suggest an exaggerated fetal response initiated by dysglycemic states in late pregnancy leading to macrosomia. These data are in keeping with the results of the Hyperglycemia and Adverse Pregnancy Outcome (HAPO) study showing associations between maternal glucose concentrations in a 75-g OGTT, even below diagnostic thresholds, and adverse pregnancy outcomes [21].

We hypothesized that dysglycemia at the end of such pregnancies might be among the factors that contribute to the association between maternal obesity at conception and later childhood overweight. The results of the mediation analysis suggested that reduction of dysglycemia by efficacious interventions in the third trimester could possibly ameliorate the weight gain of preschool children that is related to maternal pre-conception obesity by 29%. Modifying this and other early-life metabolic risks appears to be a promising target in the development of concepts for the prevention of offspring overweight.

A higher BMI in offspring of mothers with abnormal glucose regulation in pregnancy appears to take time to emerge. Follow-up studies in children up to age 2.5 years did not find a substantial impact of impaired maternal glucose metabolism in pregnancy on offspring obesity [42], and several studies have reported higher BMI *z-*scores beyond preschool age [43,44]. The emergence of an upward shift in BMI *z-*score slope and a higher weight status at a somewhat earlier age (4 years) in the offspring of obese, GDM-negative mothers with high HbA_1c_ in our study might be due to fetal exposure to an additionally obesogenic milieu in utero and an altered “glycemic threshold” in the last trimester. By contrast, the negative slope for BMI *z-*score in offspring of obese, GDM-negative mothers with normal HbA_1c_ at delivery is comparable to the BMI decline that precedes the adiposity rebound at 6 years in the general population [45] and at around 5 years in children of mothers with pre-conception obesity [46].

Since mechanisms underlying the decompensation of glucose homeostasis in late pregnancy in obese women are largely unknown, we speculated that influences associated with excessive GWG in the third trimester could play a role. Indeed, we found that obese, GDM-negative women with excessive GWG in the third trimester were at a considerably higher risk of developing dysglycemia in late pregnancy than those without excessive third-trimester GWG. Interestingly, the proportion of obese women with excessive GWG was higher in the GDM-negative than in the GDM-positive group (77.9%, 95% CI 74.0%–81.7%, versus 56.0%, 95% CI 50.3%–61.5%). Prior negative testing for GDM in obese women might have lowered their risk awareness relating to weight gain control, subsequently leading to excessive GWG in late pregnancy. In contrast to recent data suggesting that intervening at 28 weeks may be too late to improve short- and long-term outcomes [47], our findings suggest that monitoring late gestation in obese women may play a relevant role for childhood outcomes.

In extension of our previous findings on the relevance of late-pregnancy dysglycemia for later maternal health [17], we analyzed the risk of developing T2DM or prediabetes in obese women with a high HbA_1c_ at delivery despite negative GDM testing earlier in pregnancy. Based on cumulative prospective 3.5-year follow-up data from our maternal population, their prediabetes/T2DM risk was increased by 4-fold compared to those with normal glucose homeostasis throughout pregnancy. Interestingly, this risk was even higher than that reported in a meta-analysis of more than 4,000 obese, GDM-positive women (RR 2.85, 95% CI 2.21–3.69) [7]. Whether GDM treatment may have led to a lowered risk in these women is yet to be established, since evidence on the benefits of treatment for long-term maternal health is still limited to date [48].

The strengths of our study relate to the large contemporary mother–child PEACHES cohort, which is unique in the size of the population of obese mothers and the availability of trimester-specific data including HbA_1c_ at delivery as a marker of glycemic control in late pregnancy. Results are based on prospectively collected data, thus avoiding recall bias, and included a variety of confounding and exposure data from medical documents, in particular on GCTs and OGTTs during pregnancy. Outcome data were ascertained by trained medical staff. There were no relevant differences between offspring and mothers with available and missing follow-up data, suggesting negligible selection bias. A follow-up time of 4 years in offspring appeared to be sufficient to identify relevant BMI increments for prediction of overweight later on, since an increased preschool BMI is known to be highly predictive for overweight and obesity in adolescence [49]. Besides the impact of late-pregnancy dysglycemia on childhood outcomes, we were also able to demonstrate the relevance of late pregnancy as a susceptible time window for later glucometabolic health in the maternal population.

A limitation could be that high maternal HbA_1c_ at delivery only reflects late-pregnancy dysglycemia: obese, GDM-negative mothers with a high HbA_1c_ at delivery also had higher glucose concentrations—although below diagnostic cutoffs—at the time of GDM testing in the second trimester than those with a normal HbA_1c_. Therefore, we cannot rule out earlier priming effects on high offspring BMI *z-*scores at age 4 years, which might have already occurred in the second trimester. Additionally, a false-negative rate for detecting GDM of approximately one-third (18% to 40%) might be expected using the 50-g GCT (2-step procedure), due to its limited sensitivity [50–52]. However, among the obese, GDM-negative mothers, the proportion of high HbA_1c_ was similar irrespective of whether the 1-step or 2-step procedure was used, indicating that exposure group membership was not affected by the GDM test type. An analysis that excluded obese, GDM-negative mothers who underwent the 50-g GCT and developed late-pregnancy dysglycemia showed only a slightly lower difference in BMI *z-*score at age 4 years compared to the original analysis. Even though the rate of 1-step procedures across the participating recruitment departments ranged from 53% to 90%, this did not have an influence on the late-pregnancy dysglycemia in our study. There was also a change in the proportion of 1-step procedures performed over time (from initially more than 80% to about 30% at the end of recruitment) as a result of a change in coverage by the public health insurance system from the 1-step to the 2-step procedure in 2013 [53], which may have introduced misclassification bias. However, since maternal and child characteristics such as pre-conception BMI, total excessive GWG, and birth weight were not statistically noticeably different before and after the policy change, we do not anticipate that this change in practice influenced the associations found in the current study. Further, besides indicating glycemic status, HbA_1c_ variation may be prone to influences by non-glycemic factors such as ethnicity, age, and some diseases that may result in states of high or low glycation [23]. However, we obtained detailed information on factors that account for the biological variation in HbA_1c_, in particular iron deficiency anemia [54], and analytical and pre-analytical variation in HbA_1c_ measurements was low. The PEACHES cohort is a convenience sample, and we excluded women due to missing data, both of which factors may limit the generalizability of our findings. The overarching study hypotheses are outlined in the study protocol, and our results confirm these hypotheses in general. However, the specific modeling is hypothesis-generating, and its adequacy needs to be validated with more data from the PEACHES cohort. The effect found in the current study is the result of an unplanned interim analysis, and, therefore, alpha error inflation may have resulted in a higher type I error compared to the standard of 5%. However, this issue will be settled after analysis of the full dataset. The results of the interim analysis will have no influence on the further conduct of the study (such as changing follow-up or procedures); recruitment of pregnant women was completed before starting to work on this paper. Also, validation of our findings in other studies and settings is required to improve our understanding of late-pregnancy dysglycemia and its potential implications for the long-term health of the obese mother and her child.

Together, our data point to the necessity of guidelines for identifying and managing late-pregnancy complications in obese pregnancies with negative GDM testing. Screening and diagnosis of GDM is highly valuable, but dysglycemia may still arise in the last trimester, particularly in obese women. Our findings suggest that negative GDM testing at the end of the second trimester in obese pregnancies cannot be considered an “all-clear signal” and should not lead to reduced physician attention, care, and advice. Further, a false sense of security in obese, GDM-negative mothers who consider themselves unexposed to late-pregnancy dysglycemia may result in uncontrolled excessive weight gain due to unfavorable lifestyle behaviors in the last trimester. Therefore, obese women, specifically those with a negative GDM test result, require counseling on their persisting late-pregnancy risks. Tailored BMI-dependent advice including dietary therapy for late-pregnancy glycemic and weight gain control appears to be a suitable intervention. In addition, we suggest monitoring fasting glucose and HbA_1c_ at least once during the third trimester in obese women who were negative for GDM. Retesting these markers at delivery might help to identify “at risk” mother–child pairs for closer preventive health follow-ups.

## Supporting information

S1 STROBE Checklist (DOC)

S1 Fig Graph showing linear relationship between maternal HbA_1c_ at delivery and BMI *z-*score in 4-year-old offspring of obese, GDM-negative mothers. Adjusted for maternal pre-conception BMI, total gestational weight gain, maternal smoking at any time during pregnancy, and exclusive breastfeeding ≥1 month. BMI, body mass index; GDM, gestational diabetes mellitus; HbA_1c_, glycated hemoglobin.
(TIF)

S2 Fig Offspring 4-year BMI *z-*score according to maternal groups defined by pre-conception BMI, GDM status, and HbA_1c_ value at delivery. The group of GDM−, HbA_1c_+ mothers was compared with all other maternal groups using 1-way analysis of variance (ANOVA) and post hoc testing. Data are shown as median (horizontal lines within the boxes), 25th and 75th centile (lower and upper boundaries of the boxes), 1.5 times the interquartile range (whisker ends), and outliers (circles). Numerical values and dots within the boxes represent unadjusted mean 4-year BMI *z-*score of offspring. GDM status is according to the International Association of Diabetes and Pregnancy Study Groups criteria [18]. HbA_1c_ dichotomized based on a predefined cutoff value of ≥5.7% (39 mmol/mol) [17]. BMI, body mass index; GDM, gestational diabetes mellitus; HbA_1c_, glycated hemoglobin; HbA_1c_−, HbA_1c_ < 5.7% (39 mmol/mol); HbA_1c_+, HbA_1c_ ≥ 5.7%.
(TIF)

S1 Study Protocol (DOCX)

S1 Table Comparison of relevant characteristics of the study participants included and excluded from analysis due to missing data. (DOCX)

S2 Table Glucose concentrations of a 75-g OGTT at GDM testing among obese, GDM-negative mothers stratified according to their HbA_1c_ at delivery. (DOCX)

S3 Table Offspring follow-up rates at different ages in women included in the present analysis. (DOCX)

S4 Table Comparison of relevant characteristics in normal weight and obese mothers with available and missing offspring follow-up at 4 years of age. (DOCX)

S5 Table Late-pregnancy dysglycemia in obese, GDM-positive mothers and offspring outcomes. (DOCX)

S6 Table Comparison of relevant characteristics in obese, GDM-negative women with available and missing maternal follow-up visit 3.5 years postpartum. (DOCX)

## Abbreviations

BIA: bioelectrical impedance analysis
BMI: body mass index
CI: confidence interval
GCT: glucose challenge test
GDM: gestational diabetes mellitus
GWG: gestational weight gain
HbA_1c_: glycated hemoglobin
HPLC: high performance liquid chromatography
IADPSG: International Association of Diabetes and Pregnancy Study Groups
IQR: interquartile range
LGA: large-for-gestational-age
OGTT: oral glucose tolerance test
PEACHES: Programming of Enhanced Adiposity Risk in Childhood–Early Screening
RR: relative risk
SD: standard deviation
T1DM: type 1 diabetes mellitus
T2DM: type 2 diabetes mellitus
WHO: World Health Organization
