# Supplementary material for: Late-pregnancy dysglycemia in obese pregnancies after negative testing for gestational diabetes and risk of future childhood overweight: An interim analysis from a longitudinal mother–child cohort study
Source: PLoS Med. 2018 Oct 29;15(10):e1002681. doi: 10.1371/journal.pmed.1002681 (PMC6205663; doi:10.1371/journal.pmed.1002681)
Supplement: S1 STROBE Checklist — (DOC) [file pmed.1002681.s001.doc]

STROBE Statement—Checklist of items that should be included in reports of ***cohort studies***

|  | Item No | Recommendation | Author’s Response |
| --- | --- | --- | --- |
| **Title and abstract** | 1 | (*a*) Indicate the study’s design with a commonly used term in the title or the abstract | Title and Abstract, “Methods and findings”. |
|  |  | (*b*) Provide in the abstract an informative and balanced summary of what was done and what was found | Abstract, “Background” and “Methods and findings”. |
| Introduction | | |  |
| Background/rationale | 2 | Explain the scientific background and rationale for the investigation being reported | Introduction, paragraphs 1 to 4. |
| Objectives | 3 | State specific objectives, including any prespecified hypotheses | Introduction, paragraph 4. |
| Methods | | |  |
| Study design | 4 | Present key elements of study design early in the paper | Methods, “Study design and participants”. |
| Setting | 5 | Describe the setting, locations, and relevant dates, including periods of recruitment, exposure, follow-up, and data collection | Methods, “Study design and participants”, “Procedures”.  Fig 1. S3 Table. |
| Participants | 6 | (*a*) Give the eligibility criteria, and the sources and methods of selection of participants. Describe methods of follow-up | Methods, “Study design and participants”, “Inclusion criteria for analysis”, “Outcome variables”.  S1 Table. |
|  |  | (*b*) For matched studies, give matching criteria and number of exposed and unexposed | Not available |
| Variables | 7 | Clearly define all outcomes, exposures, predictors, potential confounders, and effect modifiers. Give diagnostic criteria, if applicable | Methods, “Procedures”. |
| Data sources/ measurement | 8* | For each variable of interest, give sources of data and details of methods of assessment (measurement). Describe comparability of assessment methods if there is more than one group | Methods, “Procedures”. |
| Bias | 9 | Describe any efforts to address potential sources of bias | Methods, “Exposure variables” and “Statistical analysis”. Discussion, paragraph 8-9.  S1 Table, S4 Table, S6 Table. |
| Study size | 10 | Explain how the study size was arrived at | Results, “Study population”. Fig 1, Table 1, S1 Table, S3 Table,  S4 Table, S6 Table. |
| Quantitative variables | 11 | Explain how quantitative variables were handled in the analyses. If applicable, describe which groupings were chosen and why | Methods, “Procedures”.  Results, “Study population”. Table 1. |
| Statistical methods | 12 | (*a*) Describe all statistical methods, including those used to control for confounding | Methods, “Statistical analysis”. |
|  |  | (*b*) Describe any methods used to examine subgroups and interactions | Methods, “Statistical analysis”. |
|  |  | (*c*) Explain how missing data were addressed | Methods, “Inclusion criteria for analysis”, “Statistical analysis”. Results, “Study population”.  Discussion, paragraph 8/9.  Table 1, footnote. Table 4, footnote.  Fig 1, S1 Table, S3 Table, S4 Table, S6 Table. |
|  |  | (*d*) If applicable, explain how loss to follow-up was addressed | Any losses to follow-up were excluded from analysis.  Methods, “Statistical analysis”.  Figure 1, footnote. Table 4, footnote.  S3 Table, S4 Table, S6 Table. |
|  |  | (*e*) Describe any sensitivity analyses | We conducted different subgroup analyses to study the robustness of our results.  Confirmatory analysis: Results, “Late-pregnancy dysglycemia in obese, GDM-negative women and their future diabetes risk”. Table 4. Discussion, paragraph 7 and 9. |
| Results | | |  |
| Participants | 13* | (a) Report numbers of individuals at each stage of study—eg numbers potentially eligible, examined for eligibility, confirmed eligible, included in the study, completing follow-up, and analysed | Results, “Study population” and “Late-pregnancy dysglycemia and longitudinal offspring outcomes in early childhood”.  Fig 1, Fig 2. Tables 1 to 4. S1 to S6 Tables. S2 Fig. |
|  |  | (b) Give reasons for non-participation at each stage | Fig 1, Table 4, footnote.  S1 Table, S3 Table, S4 Table, S6 Table. |
|  |  | (c) Consider use of a flow diagram | Fig 1. |
| Descriptive data | 14* | (a) Give characteristics of study participants (eg demographic, clinical, social) and information on exposures and potential confounders | Results, “Study population”,  Table 1, S1 Table, S4 Table,  S6 Table. |
|  |  | (b) Indicate number of participants with missing data for each variable of interest | Number of participants with complete data are given in the following figures and tables:  Fig 1, Fig 2. Tables 1 to 4. S2 Fig.  S1 to S6 Tables.  Participants with any missing data were excluded from analysis. |
|  |  | (c) Summarise follow-up time (eg, average and total amount) | Methods, “Offspring weight and metabolic outcomes”.  Results, “Prenatal risk factors for increased childhood weight status”, “Late-pregnancy dysglycemia in obese, GDM-negative women and their future diabetes risk”.  Tables 1 to 4, Fig 1, S3 Table.  S5 Table. |
| Outcome data | 15* | Report numbers of outcome events or summary measures over time | Results, “Prenatal risk factors for increased childhood weight status”.  Tables 2 to 4. Fig 2 and Fig 3.  S1 and S5 Table. S2 Fig. |
| Main results | 16 | (*a*) Give unadjusted estimates and, if applicable, confounder-adjusted estimates and their precision (eg, 95% confidence interval). Make clear which confounders were adjusted for and why they were included | Methods, “Statistical analysis”.  Results, “Prenatal risk factors for increased childhood weight status”,  “Late-pregnancy dysglycemia and longitudinal offspring outcomes in early childhood”,  “Excessive weight gain and deterioration of glucometabolic control in the last trimester following negative GDM testing”,  “Late-pregnancy dysglycemia in obese, GDM-negative women and their future diabetes risk”.  Table 2 (footnote), Table 3 (footnote), Table 4 (footnote), Fig 3 (footnote),  S1 Fig (footnote), S5 Table (footnote). |
|  |  | (*b*) Report category boundaries when continuous variables were categorized | Methods, “Exposure variables” and “Offspring weight and metabolic outcomes”. |
|  |  | (*c*) If relevant, consider translating estimates of relative risk into absolute risk for a meaningful time period | Results, “Late-pregnancy dysglycemia in obese, GDM-negative women and their future diabetes risk” |
| Other analyses | 17 | Report other analyses done—eg analyses of subgroups and interactions, and sensitivity analyses | We conducted different subgroup analyses to study the robustness of our results.  Mediation analysis: Results,  “Late-pregnancy dysglycemia and longitudinal offspring outcomes in early childhood”, Fig 3.  Confirmatory analysis: Results, “Late-pregnancy dysglycemia in obese, GDM-negative women and their future diabetes risk”, Table 4.  Discussion, paragraph 7 and 9. |
| Discussion | | |  |
| Key results | 18 | Summarise key results with reference to study objectives | Discussion, paragraph 1. |
| Limitations | 19 | Discuss limitations of the study, taking into account sources of potential bias or imprecision. Discuss both direction and magnitude of any potential bias | Discussion, paragraph 9. |
| Interpretation | 20 | Give a cautious overall interpretation of results considering objectives, limitations, multiplicity of analyses, results from similar studies, and other relevant evidence | Discussion, paragraphs 2 to 3, 5 to 7, 9. |
| Generalisability | 21 | Discuss the generalisability (external validity) of the study results | Discussion, paragraph 9. |
| Other information | | |  |
| Funding | 22 | Give the source of funding and the role of the funders for the present study and, if applicable, for the original study on which the present article is based | Submission form, information in “Financial Disclosure” field. |

*Give information separately for exposed and unexposed groups.

**Note:** An Explanation and Elaboration article discusses each checklist item and gives methodological background and published examples of transparent reporting. The STROBE checklist is best used in conjunction with this article (freely available on the Web sites of PLoS Medicine at http://www.plosmedicine.org/, Annals of Internal Medicine at http://www.annals.org/, and Epidemiology at http://www.epidem.com/). Information on the STROBE Initiative is available at http://www.strobe-statement.org.
