# Supplementary figures and images for: Late-pregnancy dysglycemia in obese pregnancies after negative testing for gestational diabetes and risk of future childhood overweight: An interim analysis from a longitudinal mother–child cohort study
Source: PLoS Med. 2018 Oct 29;15(10):e1002681. doi: 10.1371/journal.pmed.1002681 (PMC6205663; doi:10.1371/journal.pmed.1002681)

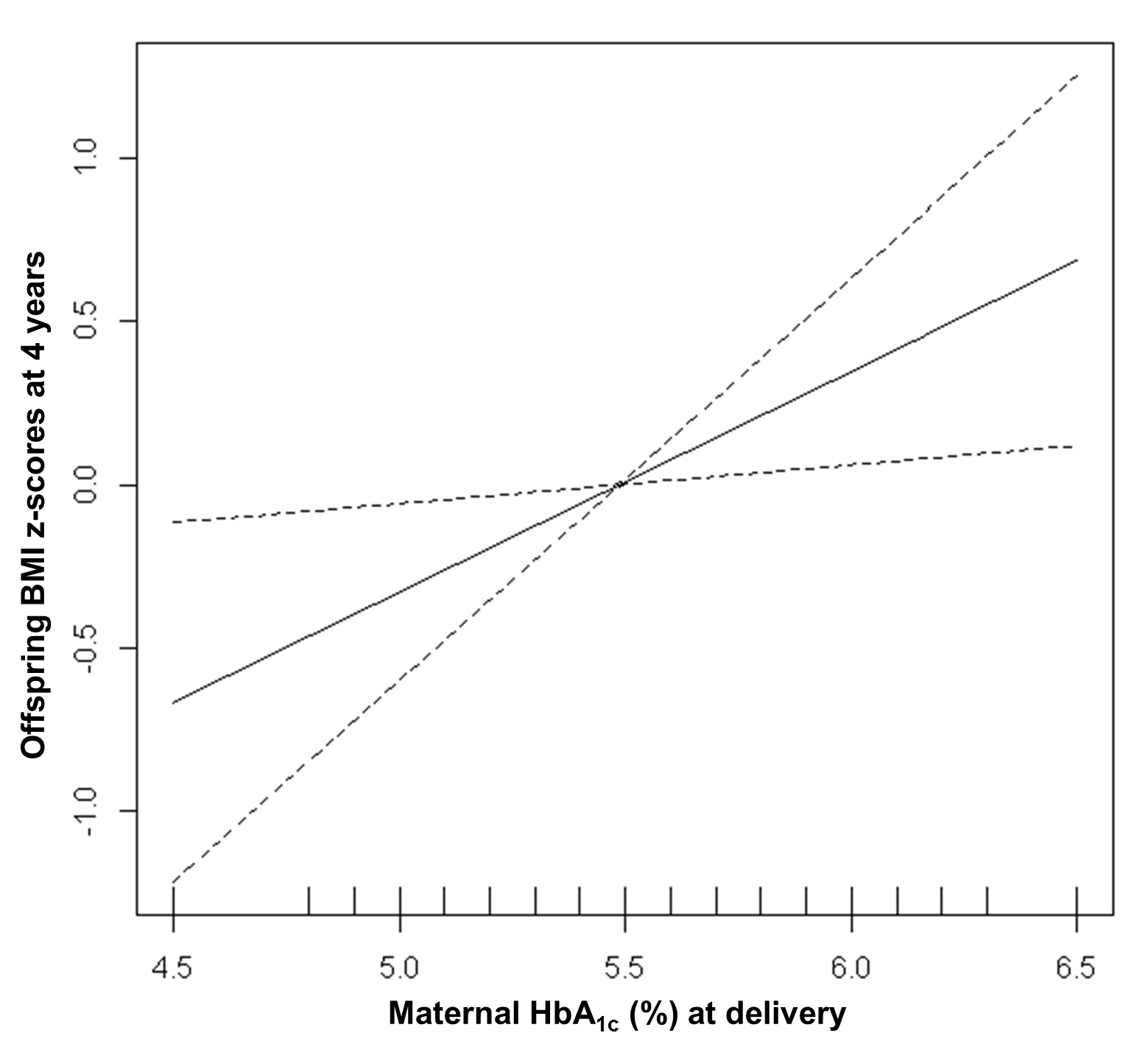

Supplement: S1 Fig — Adjusted for maternal pre-conception BMI, total gestational weight gain, maternal smoking at any time during pregnancy, and exclusive breastfeeding ≥1 month. BMI, body mass index; GDM, gestational diabetes mellitus; HbA1c, glycated hemoglobin. (TIF) [file pmed.1002681.s002.tif]

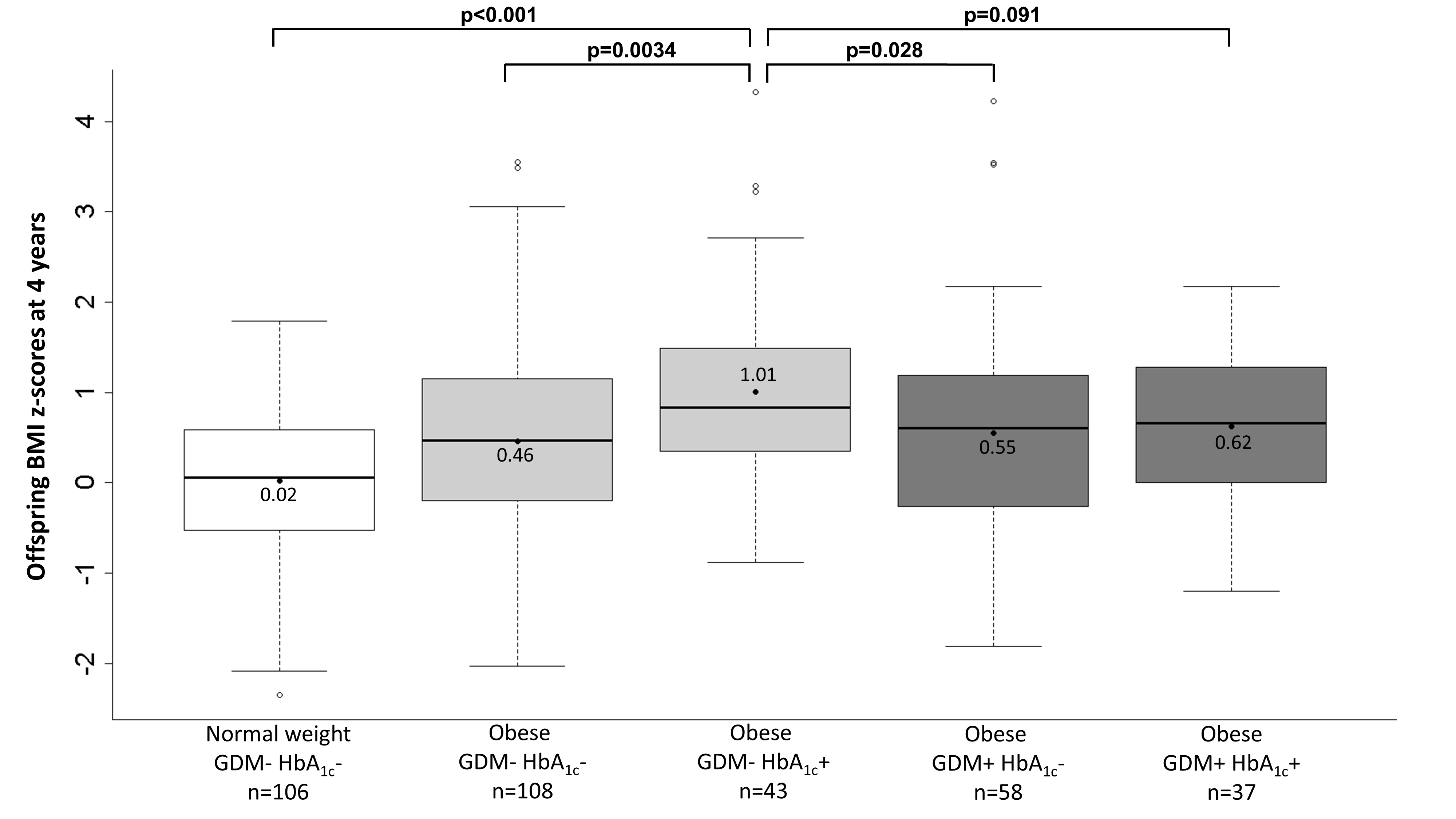

Supplement: S2 Fig — The group of GDM−, HbA1c+ mothers was compared with all other maternal groups using 1-way analysis of variance (ANOVA) and post hoc testing. Data are shown as median (horizontal lines within the boxes), 25th and 75th centile (lower and upper boundaries of the boxes), 1.5 times the interquartile range (whisker ends), and outliers (circles). Numerical values and dots within the boxes represent unadjusted mean 4-year BMI z-score of offspring. GDM status is according to the International Association of Diabetes and Pregnancy Study Groups criteria [18]. HbA1c dichotomized based on a predefined cutoff value of ≥5.7% (39 mmol/mol) [17]. BMI, body mass index; GDM, gestational diabetes mellitus; HbA1c, glycated hemoglobin; HbA1c−, HbA1c < 5.7% (39 mmol/mol); HbA1c+, HbA1c ≥ 5.7%. (TIF) [file pmed.1002681.s003.tif]
