# Supplementary material for: Late-pregnancy dysglycemia in obese pregnancies after negative testing for gestational diabetes and risk of future childhood overweight: An interim analysis from a longitudinal mother–child cohort study
Source: PLoS Med. 2018 Oct 29;15(10):e1002681. doi: 10.1371/journal.pmed.1002681 (PMC6205663; doi:10.1371/journal.pmed.1002681)
Supplement: S1 Study Protocol — (DOCX) [file pmed.1002681.s004.docx]

**Study protocol: Does childhood overweight start *in utero*?**Prospective study on “at risk” children of obese mothers to evaluate candidate markers for adiposity risk

**PEACHES: P**rogramming of **E**nhanced **A**diposity Risk in **CH**ildhood – **E**arly **S**creening

**Project lead:** Regina Ensenauer

**Background**

The prevalence of overweight and obesity among children and adolescents has increased over the last two decades and has become a major public health problem [1,2]. According to the results of the German Health Interview and Examination Survey for Children and Adolescents (KiGGS), 15% of children and adolescents in Germany are overweight and 6.3% are obese [3]. Current treatment methods to reduce obesity and to mitigate complications such as insulin resistance and dyslipidemia are not considered to be successful [4], which emphasizes the importance of the development of obesity prevention concepts.

Evidence for new approaches for primary prevention has emerged from epidemiological and animal studies, suggesting that certain environmental factors during early childhood and the maternal milieu *in utero* during fetal development may have long-term consequences for human health throughout the life span. A multitude of studies have found relationships between fetal development and later risk for diseases such as diabetes or cardiovascular problems in adulthood [5-10]. In addition, there is evidence for an association between the development of overweight/obesity and metabolic influences on fetal development via poorly defined mechanisms called "fetal programming" [11,12]. Scientific data suggest that an increased pre-pregnancy maternal body mass index (BMI) is a risk factor for later childhood overweight. Additionally, gestational diabetes mellitus (GDM), which occurs more often in obese pregnant women, increases the child’s risk of becoming overweight and developing diabetes later in life. Recent data also suggest a potential “programming” effect of maternal weight gain during pregnancy on the fetal metabolic environment. Moreover, maternal obesity and GDM place the mother at a higher risk for adverse health outcomes such as type 2 diabetes or metabolic syndrome (abdominal obesity, elevated triglyceride levels, decreased HDL cholesterol levels, increased blood pressure, elevated fasting glucose levels) [13].

A number of biomarkers are already established for the biochemical characterization of overt obesity [14]. However, these do not permit any or only very limited conclusions regarding obesity risk. Such new "risk" biomarkers (e.g. metabolites) are currently not available but are essential for the safe management of early-onset nutritional prevention strategies in future clinical trials. In the prospective mother-child cohort study PEACHES, biomarker candidates for the early detection of obesity risk will be evaluated in "high-risk" newborns of obese mothers, who will have longitudinal follow-up throughout early childhood until the age of 5 years. Further, as a subproject of the PEACHES study, mothers with an increased “gestational risk profile” such as obesity will be evaluated for cardiometabolic dysfunction several years postpartum.

**Hypotheses and Aims**

Hypotheses:

1. Overt obesity is defined by systemic-metabolic dysregulation in the intermediary metabolism.
2. The effects of metabolic dysregulation before and/or during pregnancy are associated with the risk of subsequent overweight/obesity in the child.
3. The metabolic dysregulation that is associated with the risk of offspring overweight/obesity cannot be detected clinically at birth or in early life.
4. Changes in biomarker profiles in the child’s cells represent this systemic dysregulation of the intermediary metabolism at birth or in early life as an indicator for the risk of obesity.

Aim:
The aim is to derive early prognostic biomarker(s) for childhood obesity risk.

**Study population**

PEACHES is a prospective cohort study with two different study groups and two control groups. Study group 1 consists of obese women (pre-pregnancy BMI ≥ 30 kg/m²) without GDM and study group 2 consists of obese women with GDM. Accordingly, control group 1 consists of normal weight women (pre-pregnancy BMI ≥ 18.5 ≤ 24.9 kg/m²) without GDM, while control group 2 consists of normal weight women with GDM.

**Sample size**

The sample size for the PEACHES study was calculated by Prof. Dr. Ulrich Mansmann, Institute for Medical Information Processing, Biometry, and Epidemiology (IBE), Faculty of Medicine, Ludwig-Maximilians-Universität München, Munich, Germany.

Assumptions for the calculation:

- The prevalence of obesity in pre-school children of obese mothers (BMI at conception ≥ 30 kg/m^2^) is 23% [15,16].
- The odds ratio for the offspring to be obese (versus not obese) is 3. This strong effect was based on previous findings from various studies.
- The significance level was set at 5% and the power at 80%.

Assuming that 20% of children of obese mothers with a dysregulated biomarker profile and 20% of children of obese mothers with a non-dysregulated biomarker profile are at risk of developing obesity, *n*=184 obese pregnant women should be included in the study. Assuming that 40% of children of obese mothers with a dysregulated biomarker profile and 15% of children of obese mothers with a non-dysregulated biomarker profile are at risk of developing obesity, *n*=157 obese women should be included in the study. Assuming a 30 to 40% drop-out rate up to the examination time point at age 5 years, 300 obese pregnant women (study group 1) should be recruited. In addition, 30 normal weight healthy controls (control group 1) will also be recruited. This will facilitate the assessment of the prenatal influence of maternal obesity on a possible dysregulation of the biomarker panel at birth.

For the calculation of the study group of obese mothers with GDM, the same assumptions are made. It is therefore necessary to recruit an additional 300 obese mothers with GDM (study group 2) and 30 normal weight women with GDM (control group 2).

**Examination schedule**

Children will be examined at birth, at 3 to 4 months and 5 years of age. A study questionnaire will be mailed to the families 6 to 8 weeks after birth and then annually thereafter until age 5 years. Mothers will be examined 3 years postpartum.

**Variables**

**Exposure variables.** Maternal pre-pregnancy obesity, GDM status, and metabolic risk markers such as maternal HbA_1c_ at delivery and offspring metabolites in cord blood.

**Outcome variables.** Offspring outcomes at birth: Birth weight, large-for-gestational-age (LGA) birth weight, cord-blood C-peptide concentration.

Offspring longitudinal weight status: Offspring age- and sex-specific BMI *z*-score at follow-up visits and well-child visits, as well as waist circumference at 5 years.

Maternal postpartum follow-up: Development of prediabetic/diabetic conditions based on maternal HbA_1c_ and glucose concentrations following an oral glucose tolerance test, body weight, height, fat mass, waist and hip circumferences, blood pressure, intima media thickness, pulse wave analysis, and genetic markers.

**Other variables.** Other maternal and child information that is collected includes socioeconomic status, maternal health and risk factors during pregnancy (such as smoking, pregnancy weight gain, hypertension), maternal health behaviors postpartum, breastfeeding, and the child’s health behaviors including nutritional status.

**Statistical analysis**

- Descriptive analysis of baseline characteristics relating to maternal and offspring factors will be conducted using Student’s *t* test for continuous and chi-square test for categorical variables.
- Univariable and multivariable linear and logistic regression models will be used to estimate the associations between risk markers and continuous and categorical outcomes such as BMI, LGA birth weights, childhood overweight, and maternal prediabetic/diabetic conditions postpartum.
- Multivariable logistic regression models will be used to predict the categorical outcomes from candidate markers such as maternal HbA_1c_ at delivery; variable selection will be done using shrinkage methods. Diagnostic properties of the models for the prediction of the outcomes from candidate markers and other variables will be assessed using receiver operating characteristic (ROC) analysis with k-fold cross validation.

All analyses will be conducted in the statistical software package R.

**Sources of funding**

The study is supported by the German Federal Ministry of Education and Research grant 01EA1307 to Regina Ensenauer and the Foundation for Cardiovascular Prevention in Childhood, Ludwig-Maximilians-Universität München, Munich, Germany.

**References**

1. Ebbeling CB, Pawlak DB, Ludwig DS. Childhood obesity: public-health crisis, common sense cure. Lancet. 2002;360(9331):473-82.

2. Lobstein T, Frelut ML. Prevalence of overweight among children in Europe. Obes Rev. 2003;4(4):195-200.

3. Moss A, Wabitsch M, Kromeyer-Hauschild K, Reinehr T, Kurth BM. [Prevalence of overweight and adiposity in German school children]. Bundesgesundheitsblatt, Gesundheitsforschung, Gesundheitsschutz. 2007;50(11):1424-31.

4. Poirier P, Despres JP. [Impact of obesity in contemporary cardiology]. Med Sci. 2005;21 Spec No:3-9.

5. Barker DJ. In utero programming of chronic disease. Clin Sci. 1998;95(2):115-28.

6. Shaheen SO, Sterne JA, Montgomery SM, Azima H. Birth weight, body mass index and asthma in young adults. Thorax. 1999;54(5):396-402.

7. Karter AJ, Rowell SE, Ackerson LM, Mitchell BD, Ferrara A, Selby JV, et al. Excess maternal transmission of type 2 diabetes. The Northern California Kaiser Permanente Diabetes Registry. Diabetes Care. 1999;22(6):938-43.

8. Innes KE, Byers TE, Marshall JA, Baron A, Orleans M, Hamman RF. Association of a woman's own birth weight with subsequent risk for gestational diabetes. JAMA. 2002;287(19):2534-41.

9. Fowden AL, Giussani DA, Forhead AJ. Intrauterine programming of physiological systems: causes and consequences. Physiology. 2006;21:29-37.

10. Catalano PM, Presley L, Minium J, Hauguel-de Mouzon S. Fetuses of obese mothers develop insulin resistance in utero. Diabetes Care. 2009;32(6):1076-80.

11. Oken E, Gillman MW. Fetal origins of obesity. Obesity research. 2003;11(4):496-506.

12. Catalano PM, Ehrenberg HM. The short- and long-term implications of maternal obesity on the mother and her offspring. BJOG. 2006;113(10):1126-33.

13. Vrachnis N, Augoulea A, Iliodromiti Z, Lambrinoudaki I, Sifakis S, Creatsas G. Previous gestational diabetes mellitus and markers of cardiovascular risk. Int J Endocrinol. 2012;2012:458610.

14. Mansour AA, Al-Jazairi MI. Cut-off values for anthropometric variables that confer increased risk of type 2 diabetes mellitus and hypertension in Iraq. Arch Med Res. 2007;38(2):253-8.

15. Li C, Kaur H, Choi WS, Huang TT, Lee RE, Ahluwalia JS. Additive interactions of maternal prepregnancy BMI and breast-feeding on childhood overweight. Obesity research. 2005;13(2):362-71.

16. Reilly JJ, Armstrong J, Dorosty AR, Emmett PM, Ness A, Rogers I, et al. Early life risk factors for obesity in childhood: cohort study. BMJ (Clinical research ed). 2005;330(7504):1357.
