# Supplementary material for: Late-pregnancy dysglycemia in obese pregnancies after negative testing for gestational diabetes and risk of future childhood overweight: An interim analysis from a longitudinal mother–child cohort study
Source: PLoS Med. 2018 Oct 29;15(10):e1002681. doi: 10.1371/journal.pmed.1002681 (PMC6205663; doi:10.1371/journal.pmed.1002681)
Supplement: S1 Table — (DOCX) [file pmed.1002681.s005.docx]

| S1 Table: Comparison of relevant characteristics of the study participants included and excluded from analysis due to missing data. | | | | | |
| --- | --- | --- | --- | --- | --- |
|  | **Included (n=898)** | | **Excluded due to missing data**^a^ **(n=259)** | | ***p*-value** |
|  | *N* |  | *N* |  |  |
| **Maternal characteristics during pregnancy** | | | | | |
| Pre-conception BMI, kg/m^2 b^ | 896 | 34.3 (7.4) | 244 | 34.3 (7.6) | 1 |
| Fasting glucose at GDM testing, mmol/l^c^ | 630 | 4.88 (0.80) | 117 | 5.03 (0.83) | 0.080 |
| GDM diagnosis: negative | 898 | **597 (66.5%)** | 148 | **82 (55.4%)** | **0.012** |
| Smoking at any time during pregnancy | 898 | 227 (25.3%) | 254 | 63 (24.8%) | 0.94 |
| **Maternal characteristics at delivery** | | | | | |
| Total GWG, kg | 898 | **12.6 (7.5)** | 239 | **11.4 (7.1)** | **0.022** |
| Excessive third-trimester GWG | 896 | 591 (66.0%) | 241 | 153 (63.5%) | 0.52 |
| Third-trimester GWG, kg | 896 | 4.7 (3.5) | 248 | 4.7 (3.9) | 0.84 |
| HbA_1c_ at delivery, percent^d^ | 898 | 5.50 (0.39) | 176 | 5.53 (0.36) | 0.41 |
| HbA_1c_ ≥5.7% at delivery | 898 | 271 (30.2%) | 176 | 56 (31.8%) | 0.73 |
| **Child** **characteristics at birth** |  |  |  |  |  |
| Sex: female | 898 | 435 (48.4%) | 259 | 125 (48.3%) | 1 |
| Birth weight, g | 898 | 3,502 (468) | 259 | 3,499 (505) | 0.92 |
| Birth weight: LGA | 898 | 91 (10.1%) | 259 | 22 (8.5%) | 0.68 |
| Breastfeeding (exclusive), ≥1 month | 898 | 492 (54.8%) | 242 | 124 (51.2%) | 0.36 |
| BMI z-score at 4 years | 352 | 0.43 (1.08) | 144 | 0.49 (1.05) | 0.53 |
| Data are mean (SD) or *n* (%), Student’s *t* test for continuous and χ^2^ test for categorical variables. Bold font indicates *p* < 0.05. Participants with any missing information for baseline characteristics were excluded.  ^a^Missing data in at least one of the following variables including pre-conception BMI group (normal weight or obese), GDM status (GDM-negative or GDM-positive), maternal HbA_1c_ at delivery (<5.7% [39 mmol/mol] or ≥5.7%), or confounding variables.  ^b^Two women with missing pre-conception BMI (kg/m^2^) but available pre-conception BMI group (normal weight or obese) were included in the analyses.  ^c^GDM testing was performed at median 25 weeks and 3 days of gestation (interquartile range 3 weeks and 4 days). To convert glucose mmol/l to mg/dl, multiply by 18.018.  ^d^To convert HbA_1c_ percent to mmol/mol: IFCC HbA_1c_ unit (mmol/mol) = [10.93 × DCCT/NGSP unit (%)] − 23.50.  BMI, body mass index; DCCT/NGSP, Diabetes Control and Complications Trial/National Glycohemoglobin Standardization Program; GDM, gestational diabetes mellitus; GWG, gestational weight gain; HbA_1c_, glycated hemoglobin; IFCC, International Federation of Clinical Chemistry and Laboratory Medicine; LGA, large-for-gestational-age; SD, standard deviation. | | | | | |
