# Supplementary material for: Late-pregnancy dysglycemia in obese pregnancies after negative testing for gestational diabetes and risk of future childhood overweight: An interim analysis from a longitudinal mother–child cohort study
Source: PLoS Med. 2018 Oct 29;15(10):e1002681. doi: 10.1371/journal.pmed.1002681 (PMC6205663; doi:10.1371/journal.pmed.1002681)
Supplement: S2 Table — (DOCX) [file pmed.1002681.s006.docx]

| S2 Table: Glucose concentrations of a 75-g OGTT at GDM testing among obese, GDM-negative mothers stratified according to their HbA_1c_ at delivery. | | | | |
| --- | --- | --- | --- | --- |
| **Maternal glucose concentrations** | ***N*** | **Obese, GDM−,** | ***N*** | **Obese, GDM−,** |
| **(mmol/l)**^a^ |  | **normal HbA_1c_** |  | **high HbA1c** |
| Fasting | 186 | 4.41 (4.35 to 4.47) | 80 | **4.52 (4.44 to 4.60)** |
| 1-h post-load | 185 | 7.15 (6.95 to 7.35) | 81 | **7.66 (7.39 to 7.93)** |
| 2-h post-load | 177 | 5.68 (5.52 to 5.84) | 75 | **6.14 (5.92 to 6.36)** |
| Data are mean (95% CI), and *p*-values are from Student’s *t* test. High HbA_1c_ is HbA_1c_ ≥ 5.7% (39 mmol/mol)]; normal HbA_1c_ is HbA_1c_ < 5.7%. Bold font indicates *p* < 0.05.  ^a^GDM testing was performed at median 25 weeks and 3 days of gestation (interquartile range 3 weeks and 4 days). To convert glucose mmol/l to mg/dl, multiply by 18.018.  CI, confidence interval; GDM, gestational diabetes mellitus; HbA_1c_, glycated hemoglobin; OGTT, oral glucose tolerance test. | | | | |
