# Supplementary material for: Late-pregnancy dysglycemia in obese pregnancies after negative testing for gestational diabetes and risk of future childhood overweight: An interim analysis from a longitudinal mother–child cohort study
Source: PLoS Med. 2018 Oct 29;15(10):e1002681. doi: 10.1371/journal.pmed.1002681 (PMC6205663; doi:10.1371/journal.pmed.1002681)
Supplement: S3 Table — (DOCX) [file pmed.1002681.s007.docx]

| S3 Table: Offspring follow-up rates at different ages in women included in the present analysis. | | | | | | | |
| --- | --- | --- | --- | --- | --- | --- | --- |
| **Offspring age** | **Follow-up** | **Normal weight mothers, GDM−, normal HbA_1c_** | **Obese mothers stratified by glucometabolic status during pregnancy (GDM testing) and at delivery (HbA_1c_)** | | | | **Total** |
|  |  |  | **GDM−, normal HbA_1c_** | **GDM−, high HbA_1c_** | **GDM+, normal HbA_1c_** | **GDM+, high HbA_1c_** |  |
| **2 years** | Missing^a^ | 5 (3.5%) | 13 (4.7%) | 2 (1.7%) | 5 (3.4%) | 9 (7.1%) | 34 (4.2%) |
|  | Available | 138 (96.5%) | 262 (95.3%) | 116 (98.3%) | 143 (96.6%) | 117 (92.9%) | 776 (95.8%) |
|  | Total | 143 (100%) | 275 (100%) | 118 (100%) | 148 (100%) | 126 (100%) | 810 (100%) |
| **3 years** | Missing^a^ | 9 (6.5%) | 17 (7.0%) | 7 (6.4%) | 10 (7.6%) | 9 (8.0%) | 52 (7.1%) |
|  | Available | 130 (93.5%) | 225 (93.0%) | 103 (93.6%) | 122 (92.4%) | 104 (92.0%) | 684 (92.9%) |
|  | Total | 139 (100%) | 242 (100%) | 110 (100%) | 132 (100%) | 113 (100%) | 736 (100%) |
| **4 years** | Missing^a^ | 10 (8.6%) | 17 (13.6%) | 3 (6.5%) | 5 (7.9%) | 12 (24.5%) | 47 (11.8%) |
|  | Available | 106 (91.4%) | 108 (86.4%) | 43 (93.5%) | 58 (92.1%) | 37 (75.5%) | 352 (88.2%) |
|  | Total | 116 (100%) | 125 (100%) | 46 (100%) | 63 (100%) | 49 (100%) | 399 (100%) |
| Data are *n* (%). High HbA_1c_ is HbA_1c_ ≥ 5.7% (39 mmol/mol)]; normal HbA_1c_ is HbA_1c_ < 5.7%.  ^a^Loss to follow-up or withdrawal from participation.  GDM, gestational diabetes mellitus; HbA_1c_, glycated hemoglobin. | | | | | | | |
