# Supplementary material for: Late-pregnancy dysglycemia in obese pregnancies after negative testing for gestational diabetes and risk of future childhood overweight: An interim analysis from a longitudinal mother–child cohort study
Source: PLoS Med. 2018 Oct 29;15(10):e1002681. doi: 10.1371/journal.pmed.1002681 (PMC6205663; doi:10.1371/journal.pmed.1002681)
Supplement: S4 Table — (DOCX) [file pmed.1002681.s008.docx]

| S4 Table: Comparison of relevant characteristics in normal weight and obese mothers with available and missing offspring follow-up at 4 years of age. | | | | | | | | | | | | | | | |
| --- | --- | --- | --- | --- | --- | --- | --- | --- | --- | --- | --- | --- | --- | --- | --- |
| **Offspring follow-up at 4 years** | **Normal weight mothers,**  **GDM−, normal HbA_1c_** | | | **Obese mothers stratified by glucometabolic status during pregnancy (GDM testing) and at delivery (HbA_1c_)** | | | | | | | | | | | |
|  |  |  |  | **GDM−, normal HbA_1c_** | | | **GDM−, high HbA_1c_** | | | **GDM+, normal HbA_1c_** | | | **GDM+, high HbA_1c_** | | |
|  | **Available** | **Missing**^a^ | ***p*-value** | **Available** | **Missing**^a^ | ***p*-value** | **Available** | **Missing**^a^ | ***p*-**  **value** | **Available** | **Missing**^a^ | ***p*-**  **value** | **Available** | **Missing**^a^ | ***p*-value** |
| **Maternal characteristics during pregnancy** | | | | | | | | | | | | | | | |
| *N* | 106 | 10 |  | 108 | 17 |  | 43 | 3 |  | 58 | 5 |  | 37 | 12 |  |
| Pre-conception BMI, kg/m^2^ | 21.9 (1.6) | 21.5 (1.4) | 0.43 | 36.7 (4.9) | 36.0 (3.3) | 0.48 | 36.7 (5.3) | 40.5 (7.6) | 0.48 | 36.7 (4.5) | 36.3 (4.1) | 0.89 | 39.1 (5.3) | 36.7 (5.2) | 0.18 |
| Fasting glucose at GDM testing, mmol/l^b^ | 4.36  (0.44) | 4.29  (0.60) | 0.78 | 4.37  (0.43) | 4.58  (0.33) | 0.06 | 4.51 (0.33) | 4.66  (0.07) | 0.10 | 5.21  (0.81) | 5.07  (0.50) | 0.59 | 5.67  (0.82) | 5.77  (1.51) | 0.84 |
| Smoking at any time during pregnancy | 17  (16.0%) | 1  (10.0%) | 0.96 | **22**  **(20.4%)** | **9**  **(52.9%)** | **0.010** | 8  (18.6%) | 1  (33.3%) | 1 | 17  (29.3%) | 3  (60.0%) | 0.36 | 11  (29.7%) | 4  (33.3%) | 1 |
| **Maternal characteristics at delivery** | | | | | | | | | | | | | | | |
| Total GWG, kg | 14.8 (4.4) | 15.8 (5.0) | 0.55 | 13.3 (7.5) | 14.9 (5.4) | 0.29 | 15.7 (6.2) | 11.8 (2.5) | 0.09 | 11.3 (8.6) | 12.3 (5.1) | 0.70 | 10.9 (5.7) | 10.3 (8.3) | 0.83 |
| Excessive third-trimester GWG | 55  (51.9%) | 7  (70.0%) | 0.44 | 83 (76.9%) | 14  (82.4%) | 0.85 | 39 (90.7%) | 2  (66.7%) | 0.74 | 33  (57.9%) | 4  (80.0%) | 0.62 | 22  (61.1%) | 9  (75.0%) | 0.60 |
| Third-trimester GWG, kg | 5.06  (2.21) | 5.55 (2.80) | 0.60 | 5.32  (3.39) | 7.29  (4.71) | 0.11 | 6.00 (2.92) | 6.37 (4.01) | 0.89 | 3.82  (3.64) | 5.24 (2.87) | 0.35 | 3.77  (2.94) | 3.52  (4.12) | 0.85 |
| HbA_1c_ at delivery, percent^c^ | 5.25  (0.25) | 5.31 (0.25) | 0.52 | 5.32  (0.23) | 5.31  (0.22) | 0.76 | 5.89 (0.21) | 6.03  (0.35) | 0.56 | 5.32  (0.21) | 5.40  (0.17) | 0.38 | 5.96  (0.31) | 6.23 (0.96) | 0.35 |

| S4 Table (continued): Comparison of relevant characteristics in normal weight and obese mothers with available and missing offspring follow-up at 4 years of age. | | | | | | | | | | | | | | | |
| --- | --- | --- | --- | --- | --- | --- | --- | --- | --- | --- | --- | --- | --- | --- | --- |
| **Offspring follow-up at 4 years** | **Normal weight,**  **GDM−, normal HbA_1c_** | | | **Obese mothers stratified by glucometabolic status during pregnancy (GDM testing) and at delivery (HbA_1c_)** | | | | | | | | | | | |
|  |  |  |  | **GDM−, normal HbA_1c_** | | | **GDM−, high HbA_1c_** | | | **GDM+, normal HbA_1c_** | | | **GDM+, high HbA_1c_** | | |
|  | **Available** | **Missing**^a^ | ***p*-value** | **Available** | **Missing**^a^ | ***p*-value** | **Available** | **Missing**^a^ | ***p*-**  **value** | **Available** | **Missing**^a^ | ***p*-**  **value** | **Available** | **Missing**^a^ | ***p*-value** |
| **Child characteristics at birth** | | | | | | | | | | | | | | | |
| Sex: female | 55  (51.9%) | 5  (50.0%) | 0.58 | 51  (47.2%) | 9  (52.9%) | 0.86 | 16  (37.2%) | 3  (100.0%) | 0.17 | 32  (55.2%) | 3  (60.0%) | 1 | 16  (43.2%) | 5  (41.7%) | 1 |
| Birth weight, g | 3,465  (421) | 3,608 (524) | 0.42 | 3,481  (465) | 3,454  (328) | 0.77 | 3,599  (487) | 3,620  (334) | 0.93 | 3,450  (486) | 3,782 (455) | 0.18 | 3,696  (540) | 3,719 (432) | 0.88 |
| Birth weight: LGA | 4  (3.8%) | 2  (9.5%) | 0.09 | 6  (5.6%) | 0  (0.0%) | 0.56 | 6  (14.0%) | 1  (33.3%) | 0.62 | 5  (8.6%) | 2  (40.0%) | 0.08 | 10  (27.0%) | 3  (25.0%) | 0.69 |
| Cord-blood C-peptide, ng/ml^d^ | 0.47  (0.31) | 0.52  (0.27) | 0.61 | 0.49  (0.29) | 0.62  (0.31) | 0.13 | 0.53  (0.33) | 0.64  (0.76) | 0.82 | 0.52  (0.33) | 0.70  (0.52) | 0.47 | 0.69  (0.42) | 0.75  (0.63) | 0.78 |
| Breastfeeding (exclusive), ≥1 month | 86  (81.1%) | 7  (70.0%) | 0.65 | 61  (56.5%) | 6  (35.3%) | 0.26 | 20  (46.5%) | 1  (33.3%) | 0.49 | 32  (55.2%) | 2  (40.0%) | 0.38 | 16  (43.2%) | 5  (41.7%) | 0.97 |
| Data are mean (SD) or *n* (%), Student’s *t* test for continuous and χ^2^ test for categorical variables. High HbA_1c_ is HbA_1c_ ≥ 5.7% (39 mmol/mol)]; normal HbA_1c_ is HbA_1c_ < 5.7%. Bold font indicates *p* < 0.05. Participants with any missing information for baseline characteristics were excluded.  ^a^Loss to follow-up or withdrawal from participation.  ^b^GDM testing was performed at median 25 weeks and 3 days of gestation (interquartile range 3 weeks and 4 days). To convert glucose mmol/l to mg/dl, multiply by 18.018.  ^c^To convert HbA_1c_ percent to mmol/mol: IFCC HbA_1c_ unit (mmol/mol) = [10.93 × DCCT/NGSP unit (%)] − 23.50.  ^d^To convert C-peptide ng/ml to nmol/l, multiply by 0.331.  BMI, body mass index; DCCT/NGSP, Diabetes Control and Complications Trial/National Glycohemoglobin Standardization Program; GDM, gestational diabetes mellitus; GWG, gestational weight gain; HbA_1c_, glycated hemoglobin; IFCC, International Federation of Clinical Chemistry and Laboratory Medicine; LGA, large-for-gestational-age; SD, standard deviation. | | | | | | | | | | | | | | | |
