# Supplementary material for: Late-pregnancy dysglycemia in obese pregnancies after negative testing for gestational diabetes and risk of future childhood overweight: An interim analysis from a longitudinal mother–child cohort study
Source: PLoS Med. 2018 Oct 29;15(10):e1002681. doi: 10.1371/journal.pmed.1002681 (PMC6205663; doi:10.1371/journal.pmed.1002681)
Supplement: S5 Table — (DOCX) [file pmed.1002681.s009.docx]

| S5 Table: Late-pregnancy dysglycemia in obese, GDM-positive mothers and offspring outcomes. | | | | |
| --- | --- | --- | --- | --- |
| **Child outcome** | **Control group (obese, GDM+, normal HbA_1c_)** | | **Maternal late-pregnancy dysglycemia (obese, GDM+, high HbA_1c_)** | |
|  | ***N*** | **Mean (95% CI)** | ***N*** | **Mean increment Δ (95% CI) with respect to control group** |
| At delivery^a^ |  |  |  |  |
| Birth weight, g | 165 | 3,440 (3,368 to 3,511) | 136 | 103 (−4 to 211) |
| Cord-blood C-peptide, ng/ml^b^ | 158 | 0.57 (0.51 to 0.63) | 128 | 0.04 (−0.07 to 0.14) |
| Long-term follow-up^c^: | | | | |
| BMI z-score change per year^d^ | 321 | −0.04 (−0.15 to 0.05) | 255 | −0.003 (−0.14 to 0.15) |
| BMI z-score at 4 years^e^ | 58 | 0.55 (0.24 to 0.86) | 37 | 0.07 (−0.38 to 0.51) |
| Mean increments in offspring outcomes by high maternal HbA_1c_ (≥5.7% [39 mmol/mol]) at delivery are shown relative to the obese, GDM+, normal HbA_1c_ group.  ^a^Based on linear regression models, adjusted for maternal pre-conception BMI, total gestational weight gain, maternal smoking at any time during pregnancy, and sex of the child.  ^b^To convert C-peptide ng/ml to nmol/l, multiply by 0.331.  ^c^Adjusted for maternal pre-conception BMI, total gestational weight gain, maternal smoking at any time during pregnancy, and exclusive breastfeeding ≥1 month.  ^d^Based on linear mixed-effects model.  ^e^Based on linear regression model.  BMI, body mass index; CI, confidence interval; GDM, gestational diabetes mellitus; HbA_1c_, glycated hemoglobin. | | | | |
