# Supplementary material for: Late-pregnancy dysglycemia in obese pregnancies after negative testing for gestational diabetes and risk of future childhood overweight: An interim analysis from a longitudinal mother–child cohort study
Source: PLoS Med. 2018 Oct 29;15(10):e1002681. doi: 10.1371/journal.pmed.1002681 (PMC6205663; doi:10.1371/journal.pmed.1002681)
Supplement: S6 Table — (DOCX) [file pmed.1002681.s010.docx]

| S6 Table: Comparison of relevant characteristics in obese, GDM-negative women with available and missing maternal follow-up visit 3.5 years postpartum. | | | | | | |
| --- | --- | --- | --- | --- | --- | --- |
| **Maternal follow-up visit** | **Obese, GDM−, normal HbA_1c_** | | | **Obese, GDM−, high HbA_1c_** | | |
| **3.5 years postpartum** | **Available** | **Missing**^a^ | ***p*-value** | **Available** | **Missing**^a^ | ***p*-value** |
| **Maternal characteristics during pregnancy** | | | | | | |
| *N* | 86 | 17 |  | 37 | 12 |  |
| Pre-conception BMI, kg/m^2^ | 36.6 (4.7) | 34.8 (3.7) | 0.083 | 36.4 (5.1) | 37.9 (5.5) | 0.41 |
| Fasting glucose at GDM testing, mmol/l^b^ | 4.42 (0.41) | 4.48 (0.46) | 0.69 | 4.54 (0.36) | 4.47 (0.18) | 0.45 |
| Smoking at any time during pregnancy | 16 (18.6%) | 6 (35.3%) | 0.23 | 11 (29.7%) | 4 (33.3%) | 1 |
| **Maternal characteristics at delivery** | | | | | | |
| Total GWG, kg | 12.1 (7.2) | 13.8 (6.4) | 0.35 | 13.6 (6.8) | 12.8 (8.3) | 0.76 |
| Excessive third-trimester GWG | 65 (75.6%) | 13 (7.5%) | 0.34 | 32 (86.5%) | 10 (83.3%) | 0.24 |
| Third-trimester GWG, kg | 4.9 (3.1) | 6.4 (4.8) | 0.24 | 4.9 (2.3) | 6.8 (4.4) | 0.18 |
| HbA_1c_ at delivery, percent^c^ | 5.3 (0.3) | 5.3 (0.2) | 0.72 | 5.9 (0.2) | 6.0 (0.2) | 0.10 |
| **Child characteristics at birth** | | | | | | |
| Sex: female | 36 (41.9%) | 8 (47.1%) | 0.90 | 15 (40.5%) | 7 (58.3%) | 0.46 |
| Birth weight, g | 3,481 (448) | 3,263 (570) | 0.15 | 3,536 (501) | 3,678 (440) | 0.41 |
| Birth weight: LGA | 7 (8.1%) | 0 (0%) | 0.17 | 4 (10.8%) | 2 (16.7%) | 0.54 |
| Cord-blood C-peptide, ng/ml^d^ | 0.51 (0.32) | 0.64 (0.38) | 0.22 | 0.52 (0.36) | 0.79 (0.49) | 0.10 |
| Breastfeeding (exclusive), ≥1 month | 51 (59.3%) | 7 (41.2%) | 0.35 | 13 (35.1%) | 6 (50.0%) | 0.55 |
| Data are mean (SD) or *n* (%), Student’s *t* test for continuous and χ^2^ test for categorical variables. High HbA_1c_ is HbA_1c_ ≥ 5.7% (39 mmol/mol)]; normal HbA_1c_ is HbA_1c_ < 5.7%. Participants with any missing values for baseline characteristics were excluded.  ^a^Loss to follow-up or withdrawal from participation.  ^b^GDM testing was performed at median 25 weeks and 3 days of gestation (interquartile range 3 weeks and 4 days). To convert glucose mmol/l to mg/dl, multiply by 18.018.  ^c^To convert HbA_1c_ percent to mmol/mol: IFCC HbA_1c_ unit (mmol/mol) = [10.93 × DCCT/NGSP unit (%)] − 23.50.  ^d^To convert C-peptide ng/ml to nmol/l, multiply by 0.331.  BMI, body mass index; DCCT/NGSP, Diabetes Control and Complications Trial/National Glycohemoglobin Standardization Program; GDM, gestational diabetes mellitus; GWG, gestational weight gain; HbA_1c_, glycated hemoglobin; IFCC, International Federation of Clinical Chemistry and Laboratory Medicine; LGA, large-for-gestational-age; SD, standard deviation. | | | | | | |
